# Optimized Transformation and Gene Editing of the B104 Public Maize Inbred by Improved Tissue Culture and Use of Morphogenic Regulators

**DOI:** 10.3389/fpls.2022.883847

**Published:** 2022-04-22

**Authors:** Stijn Aesaert, Lennert Impens, Griet Coussens, Els Van Lerberge, Rudy Vanderhaeghen, Laurence Desmet, Yasmine Vanhevel, Shari Bossuyt, Angeline Ndele Wambua, Mieke Van Lijsebettens, Dirk Inzé, Ellen De Keyser, Thomas B. Jacobs, Mansour Karimi, Laurens Pauwels

**Affiliations:** ^1^Department of Plant Biotechnology and Bioinformatics, Ghent University, Ghent, Belgium; ^2^VIB Center for Plant Systems Biology, Ghent, Belgium; ^3^Plant Sciences Unit, Flanders Research Institute for Agriculture, Fisheries and Food (ILVO), Melle, Belgium

**Keywords:** maize, transformation, *Agrobacterium*, morphogenic genes, tissue culture, gene editing, CRISPR/Cas9, T-DNA

## Abstract

Plant transformation is a bottleneck for the application of gene editing in plants. In *Zea mays* (maize), a breakthrough was made using co-transformation of the morphogenic transcription factors BABY BOOM (BBM) and WUSCHEL (WUS) to induce somatic embryogenesis. Together with adapted tissue culture media, this was shown to increase transformation efficiency significantly. However, use of the method has not been reported widely, despite a clear need for increased transformation capacity in academic settings. Here, we explore use of the method for the public maize inbred B104 that is widely used for transformation by the research community. We find that only modifying tissue culture media already boosts transformation efficiency significantly and can reduce the time in tissue culture by 1 month. On average, production of independent transgenic plants per starting embryo increased from 1 to 4% using *BIALAPHOS RESISTANCE* (BAR) as a selection marker. In addition, we reconstructed the BBM-WUS morphogenic gene cassette and evaluated its functionality in B104. Expression of the morphogenic genes under tissue- and development stage-specific promoters led to direct somatic embryo formation on the scutellum of zygotic embryos. However, eight out of ten resulting transgenic plants showed pleiotropic developmental defects and were not fertile. This undesirable phenotype was positively correlated with the copy number of the morphogenic gene cassette. Use of constructs in which morphogenic genes are flanked by a developmentally controlled Cre/LoxP recombination system led to reduced T-DNA copy number and fertile T0 plants, while increasing transformation efficiency from 1 to 5% using HIGHLY-RESISTANT ACETOLACTATE SYNTHASE as a selection marker. Addition of a CRISPR/Cas9 module confirmed functionality for gene editing applications, as exemplified by editing the gene *VIRESCENT YELLOW-LIKE* (*VYL*) that can act as a visual marker for gene editing in maize. The constructs, methods, and insights produced in this work will be valuable to translate the use of BBM-WUS and other emerging morphogenic regulators (MRs) to other genotypes and crops.

## Introduction

Maize (*Zea mays*) is an important experimental model for plant genetics and the most widely grown crop worldwide (Andorf et al., 2019). The advent of CRISPR/Cas9 gene editing allows efficient investigation of maize gene function (Xing et al., 2014b; Svitashev et al., 2015; Char et al., 2017). Current methods typically rely on immature embryos as explant material and either *Agrobacterium tumefaciens* or a particle gun for delivery of the CRISPR/Cas9 DNA constructs (Ishida et al., 1996; Frame et al., 2002; Raji et al., 2018). *Agrobacterium*-mediated transformation is widely preferred due to disadvantages associated with biolistics such as genome damage (Liu et al., 2019) and a higher frequency of truncated or multicopy inserts (Kausch et al., 2021). Protocols are well-established for the inbred line B104 (Frame et al., 2006; Coussens et al., 2012; Raji et al., 2018). Immature embryos are isolated and infected with the hypervirulent strain EHA101 or the derivative EHA105 (Hood et al., 1993). For CRISPR/Cas9, strains are equipped with a binary plant transformation vector that allows expression of Cas9, one or more single guide RNAs (sgRNAs) and a selection marker such as the *BIALAPHOS RESISTANCE* (*BAR*) transgene (Char et al., 2017). After callus induction using auxins, transgenic and embryogenic calli are selected using phosphinothricin (PPT) and several rounds of tissue culture. Finally, T0 plantlets are regenerated from somatic embryos (Raji et al., 2018). Using this method, T0 plants may already contain homozygous or transheterozygous edits (Lee et al., 2019). The transformation efficiency of these protocols in our hands is, however, relatively low and variable with independent T0 events per infected immature embryo ranging from 0.25 to 4.85% (Coussens et al., 2012).

Traditionally, laborious and empirical fine-tuning of auxin/cytokinin ratios has been used to optimize callus formation and somatic embryogenesis for each maize genotype. Lately, this has been complemented by the use of morphogenic regulator (MR) genes to promote somatic embryogenesis. These include *WUSCHEL* (*WUS*) controlling stem cell maintenance (Zuo et al., 2002) and *BABY BOOM* (*BBM*) controlling embryo identity (Boutilier et al., 2002). In maize, a combined expression of the maize *BBM* ortholog *ZmBBM* also known as *OVULE DEVELOPMENT PROTEIN 2* and *ZmWUS2* is a key part of optimized transformation methods that allow efficient transformation of various elite genotypes (Lowe et al., 2016). In a first report, *ZmBBM* was expressed under control of the maize *UBIQUITIN-1* promoter (pZmUBI), and *ZmWUS2* under control of the *Agrobacterium nopaline synthase* promoter (pnos) to improve transformation using maize immature embryos as explants (Lowe et al., 2016). Due to pleiotropic effects on plant development by continued expression of *ZmBBM* and *ZmWUS2*, including reduced fertility, an inducible Cre/LoxP system was used to excise the MR cassette during tissue culture (Lowe et al., 2016). In a follow-up study, the gene *PHOSPHOLIPID TRANSFER PROTEIN* (*ZmPLTP*) was identified to be specifically expressed in the scutellum epithelium, the cell layer transformed by *Agrobacterium* (Lowe et al., 2018). Expressing *ZmBBM* using pZmPLTP and *ZmWUS2* using the auxin-inducible promoter of *ZmIAA25* (pZmAXIG1) allowed direct somatic embryogenesis and halving time in tissue culture (Lowe et al., 2018). Transformation frequencies using this technology ranged from 8.7 to 96%, depending on the genotype. Moreover, the specific MR-expression alleviated the need of Cre/LoxP-mediated excision for normal plant development (Lowe et al., 2018).

Here, we investigated the use of pZmPLTP::*ZmBBM* and pZmAXIG1::*ZmWUS2* for transformation of the public inbred line B104, currently used in various academic transformation facilities. Our results show that even without use of MR genes, B104 transformation using *BAR* as a selection marker can be improved fourfold in efficiency and reduced by one month in time by adapting tissue culture media. Additional use of the MR cassette allowed improving transformation from 1 to up to 15% using *Highly Resistant ALS* (*HRA*) as a selection marker. However, despite specific expression of *ZmBBM* and *ZmWUS2*, high copy numbers are associated with pleiotropic effects, which could be circumvented by their use in conjunction with Cre/LoxP-mediated gene excision. Finally, we show that use of *ZmBBM* and *ZmWUS2* can be used in combination with CRISPR/Cas9 by editing of *VIRESCENT YELLOW-LIKE* (*VYL*). Loss of *VYL* function results in pale-yellow leaves in tissue culture without affecting further development and fertility and can act as a visual marker for gene editing in maize.

## Materials and Methods

### Plant Material

Seeds of the maize inbred line B104 used were originally obtained from the USDA National Plant Germplasm System (Accession no. PI 594047). A single maize B104 seed was placed in a pre-wetted Jiffy-7® pellet and kept in controlled greenhouse conditions (300 μE.m^−2^.s^−1^ light intensity, 16 h light, 26°C and 8 h dark, 22°C). The seedlings were transferred to 10 L pots containing controlled release fertilizer (2.0 kg/m^3^, Osmocote® Professional potting mixture, Scotts International B.V.) and moved to a larger greenhouse until grown to maturity.

### Bacterial Strains

For cloning, DH5α competent cells (Invitrogen) and *ccdB* Survival™2 cells (Invitrogen) were used. For transformation, hypervirulent disarmed *Agrobacterium* strains EHA101 (Hood et al., 1986) and EHA105 (Hood et al., 1993) were used.

### sgRNA Design

In the presence of alternative gene models for *VYL* (*Chr.9_ClpP5*) in the B73 genome annotation (v4), we targeted exon 4, which is present in all gene models and encodes the start of the conserved ClpP5 domain. We used the B73 sequence of *VYL* exon 4 and CRISPOR^1^ to select a sgRNA with predicted high specificity and efficiency. The targeted region was amplified using wild-type B104 genomic DNA and sequenced. A single nucleotide polymorphism was found, and the spacer was adapted accordingly (TCGGTGCGGAGGCCCTGTTG).

### Vector Construction

All plasmids used in this study are listed in Supplementary Table S1, primers in Supplementary Table S2 and modules produced by gene synthesis in Supplementary Table S3.

#### BAR as a Selection Marker

For initial experiments using BAR as a selection marker, the Gateway™-compatible destination vector pBbm42GW7 (Karimi et al., 2013) was used that contains the *BAR* gene under control of the double Cauliflower mosaic virus (CaMV) 35S promoter (p35S). The resulting expression vector was then transferred to EHA101 for maize transformation.

For generating the CRISPR/Cas9 construct targeting *VYL*, the Golden Gate entry clone pGG-A-pZmUBI-B was constructed by PCR amplification using pEN-L4-UBIL-R1 (Karimi et al., 2007) as a template and primers UBI_Fw and UBI_Rv. The amplified fragment was cloned into a BsaI-digested GreenGate pGGA000 entry vector (Lampropoulos et al., 2013). Next, the *Arabidopsis* codon optimized Cas9 Gateway™ entry clone pEN-L4-pZmUBI-Cas9-tG7-R1 was constructed by Golden Gate cloning using pGG-A-pZmUBI-B and the other entry clones pGG-B-linker-C, pGG-C-Cas9-D, pGG-D-linker-E, pGG-E-tG7-F (Lampropoulos et al., 2013; Decaestecker et al., 2019), and pEN-L4-AG-R1 (Houbaert et al., 2018). For cloning of the sgRNA targeting *VYL*, two complementary oligos with 4 bp overhangs (CROPGEN7 and CROPGEN8), were annealed and inserted *via* a Golden Gate reaction with BbsI (Thermo) and T4 DNA ligase (Thermo) in the Gateway™ entry clone pMR185 (attL1-attL2, Tripathi et al., 2019) containing the OsU6 promoter. Finally, the Gateway™ destination clone pBbm42GW7 (Karimi et al., 2013) was again used to combine the Cas9 module with the sgRNA module.

#### Morphogenic Genes

Based on the sequence of PHP79066 (Lowe et al., 2018), we used GeneArt (Invitrogen) to synthesize GreenGate-compatible modules and cloned in the vectors pGG-A-C (Rodrigues et al., 2021) or pGG-C000 (Lampropoulos et al., 2013) pGG-A-pZmPLTP-C, pGG-A-pZmAXIG1-C, pGG-A-ZmGLB1-C, pGG-C-ZmBBM-D, pGG-C-ZmWUS2-D, pGG-C-LoxP-ZmNLS-mScarlet-D, and pGG-C-MoCRE-D (Supplementary Table S3).

For cloning of pLAPAU9, Golden Gateway cloning was used (Karimi and Jacobs, 2021). First, Golden Gate cloning was used to create pEN-L4-pZmUBI-LoxP-ZmNLS-mScarlet-tG7-R1, pEN-L1-linker-L2, and pEN-R2-pBdEF1a-GUS-t35S-L3 and combine these with MultiSite Gateway cloning in pGGW-A-m43GW-B, and pEN-L4-pZmPLTP-ZmBBM-tG7-R1, pEN-L1-pZmAXIG1-ZmWUS2t-t35S-L2, and pEN-R2-linker-L3 were combined in pGGW-B-m43GW-C. Finally, Golden Gate cloning was used to combine the resulting vectors pGG-A-pZmUBI-LoxP-ZmNLS-mScarlet-NLS-tG7-pBdEF1a-GUS-t35S-B, pGG-B-pZmPLTP-ZmBBM-tG7-pZmAXIG1-ZmWUS2-t35S-C and pGG-C-linkerII-G with pGGBb-AG to pBb-pZmPLTP-ZmBBM-tG7-pZmAXIG1-ZmWUS2-t35S-pZmUBI-LoxP-ZmNLS-mScarlet-NLS-tG7-pBdEF1a-GUS-t35S (pLAPAU9). For the control vector, the GUS coding sequence containing the potato IV2 intron was amplified from pXBb7-SI-UBIL (Karimi et al., 2013) using CROPGEN492 and CROPGEN493 (Supplementary Table S2) and cloned in pGGC000. Subsequently, Golden Gate cloning was used to combine it with pGG-A-pBdEF1a-B, pGG-B-linker-C and pGG-D-tnos-G.

For cloning of pLAPAU6, pEN-L4-pZmPLTP-ZmBBM-tG7-R1, and pEN-L1-pZmAXIG1-ZmWUS2-t35S-L2 were recombined with pBb7m24GW to yield pBb-pZmPLTP-ZmBBM-tG7-pZmAXIG1-ZmWUS2-t35S (pLAPAU6).

#### HRA as a Selection Marker

For selection using imazapyr, we synthesized the module pSbALS::*HRA*:tStpinII based on RV012608 (Anand et al., 2016) using Life Technologies Europe BV. This module contains the *Sorghum bicolor ALS* promoter driving the maize *HRA* gene that contains two point mutations (Green et al., 2009) and the 3′ control region of the potato *Proteinase Inhibitor II* gene (An et al., 1989). To facilitate downstream cloning, all *BbsI*, *Esp3I*, *ApaI*, *HindIII*, and *EcoRV* sites were mutated, and 30 bp homologous to pPZ200 for Gibson Assembly were added. This module was cloned with Gibson assembly to generate pRA-AG and pRA-U1-AG-U9. For the control vector pRA-ZmUBI-GUS-35ST, pGG-A-pZmUBI-B, pGG-B-linker-C, pGG-C-GUS-D, and pGG-D-t35S-G were recombined in pRA-AG.

For cloning of pLAPAU14, we used Golden Gibson cloning (Jacobs and Karimi, unpublished). Using Golden Gate cloning we combined elements into the shuttle vectors pGGIB-U3-AG-U4 and pGGIB-U6-AG-U7 to yield pGGIB-U3-pZmAXIG1-ZmWUS2-t35S-U4 and pGGIB-U6-pZmPLTP-ZmBBM-tnos-U7. In these vectors, unique nucleotide sequence (U-sites, Torella et al., 2014) of 40 bp are flanked with the I-SceI restriction sites. Using Gibson assembly, the fragments were combined in pRA-U1-AG-U9 with pGGIB-U1-linker-U3, pGGIB-U4-linker-U6, and pGGIB-U7-linker-U9 to yield pRA-pZmAXIG1-ZmWUS2-tG7-pZmPLTP-ZmBBM-tnos (pLAPAU14).

For pLAPAU16, a similar strategy was used to create pGGIB-U1-pBdEF1a.2-ATG-LoxP-G7t-U2, pGGIB-U2-pZmGLB1-MoCre-tnos-U3, pGGIB-U3-pZmAXIG1-ZmWUS2-t35S-U4, pGGIB-U4-pZmPLTP-ZmBBM-tnos-U5, pGGIB-U5-LoxP-mRUBY3-tocs, and pGGIB-U6-linker-U9 and combine these to pLAPAU16. To create pGGIB-U5-LoxP-mRUBY3-tocs, first an mRuby-ATG entry vector in pGG-C000 was constructed by PCR with mRuby lacking a start codon. The Lox fragment was designed with three stop codons flanked to the A and C GreenGate overhang sequences. The forward and reversed oligos (B1_A-LOX_F, B1_C-LOX_R) were annealed and used in a Golden Gate reaction with pGG-C-mRuby-ATG-D, pGG-D-tocs-G were cloned into Gibson entry clone pGGIB-U5-A-ccdB-G-U6 to generate pGGIB-U5-LoxP-mRUBY3-tocs.

For pLAPAU17, the modules from pGGIB-U6-pZmUBI.2-zCas9-tG7-U7, pGGIB-U7-pOsU3-BsaI-ccdB/GmR-BsaI-U8, and pGGIB-U8-linker-U9 were further included to yield pLAPAU17. For cloning of the *VYL* spacer in pLAPAU17, the oligos CROPGEN27 and CROPGEN8 (Supplementary Table S2) were annealed and cloned using BsaI.

### Maize Transformation

Immature embryos of maize inbred line B104 were transformed using *Agrobacterium* as previously described (Coussens et al., 2012) with modifications. Briefly, when immature embryos were 1.5–2 mm in size (12–14 days after fertilization), at least three ears from individual B104 plants were collected, stored overnight at 4°C and surface-sterilized the next morning. Immature embryos were isolated from the ears and co-cultivated with *Agrobacterium* for 3 days on co-cultivation medium (Supplementary Table S4) at 21°C in the dark, with the scutellum side up. After co-cultivation, embryos were transferred to non-selective resting medium (Supplementary Table S4) and incubated for 6 days in the dark at 25°C. Compared to Coussens et al. (2012), co-cultivation and resting media contain 15 μM of the auxin dicamba (Sigma-Aldrich, Saint Louis, Missouri, United States) instead of 2,4-D.

For the experiments, all following steps are identical to Coussens et al. (2012), except for 15 μM dicamba in selection I (Supplementary Table S4) and selection II media (referred to as method 1). For all experiments from 2020 and with morphogenic genes (referred to as method 2), we reduced the time on selection I from 14 to 7 days (Supplementary Tables S4**,**S5). Next, we transferred embryos on a first maturation medium for 14 days containing cupric sulfate (CuSO_4_) and the plant growth regulators IAA, thidiazuron, ABA, BAP, and zeatin (Supplementary Table S4), followed by 14 days on second maturation medium containing CuSO_4_, IAA, ABA, and BAP in the light (Supplementary Table S4). During the last tissue culture step (14 days on regeneration II medium, incubated at 25°C), plantlets develop roots. The rooted plantlets are then transferred to soil (a pre-wetted Jiffy-7 pellet) and covered with plastic box to maintain high humidity; this facilitated transition from tissue culture to soil. Transgenic plantlets were kept in controlled growth room conditions (300 μE.m^−2^.s^−1^ light intensity, 16 h light, 26°C and 8 h dark, 22°C). Humidifying cover was removed after 3 days. After 2 weeks on soil, plants were genotyped for presence of T-DNA and, in case of CRISPR/Cas9 mutagenesis, for mutations in the target gene(s). Plants that showed the desired genotype were transferred to 10 L pots containing controlled-release fertilizer (Osmocote, 2.0 kg/m^3^) and grown in the greenhouse. Resistance to PPT was assayed using AgraStrip® lateral flow strips (Romer Labs). Histochemical 5-bromo-4-chloro-3-indolyl-β-D-glucuronide (X-Gluc) assays were performed as described (Coussens et al., 2012). A VHX-7000 digital microscope (Keyence International, Mechelen, Belgium) was used for imaging of tissue culture at different stages up to regeneration II.

### Genotyping

For genomic DNA extraction, a piece of approximately 1 cm^2^ was cut from a leaf and put in a 2 ml reaction tube together with two 4 mm metal grinding beads and snap-frozen in liquid nitrogen. The tissue was then crushed using a Retsch® Mixer Mill MM 400 machine at 20 Hz for 1 min. Genomic DNA (gDNA) was isolated from the crushed, frozen leaf material using the Wizard® Genomic DNA Purification kit (Promega). gDNA samples were subjected to PCR amplification of the targeted region, by using the GoTaq® Flexi kit (Promega). PCR products were subjected to a purification step using magnetic beads (HighPrep™ PCR Clean-up System, Magbio). A Mix2Seq Kit (Eurofins Genomics) was used for sequencing of the purified PCR products and analyzed using ICE (Synthego). For genotyping of the *HRA* marker, the primers CROPGEN597 and CROPGEN598 (Supplementary Table S2) were used that target *HRA* and the *StPinII* terminator, respectively. For genotyping of *VYL*, CROPGEN6, and CROPGEN56 were used, for *Chr.1_ClpP5* CROPGEN452, and CROPGEN453 were used (Supplementary Table S2).

### Droplet Digital PCR

Droplet digital PCR (ddPCR) was used as previously described (Desmet et al., 2020) for gene copy number measurements. Reference primers for the single-copy genes *FPGS* (Zm00007a00000670, Manoli et al., 2012) and *ADH1* (Collier et al., 2017) were used. Additional primers were designed for p*ZmPLTP*::*ZmBBM*, t*StPinII* (HRA terminator), and *aadA* (backbone marker) and are listed in Supplementary Table S2. Genomic DNA of T0 plants was isolated using the Wizard® Genomic DNA Purification kit (Promega), quantified using the Qubit dsDNA HS Assay Kit (Thermo) and digested with CviQI (NEB) for 1 h. Per sample, 15 ng of digested gDNA was used as input for ddPCR with ddPCR EvaGreen supermix (BioRad) and analyzed using the QX200™ (BioRad) in duplicate with a ramp rate of 1°C/s. ddpcRquant (Trypsteen et al., 2015) was used for calculating copy numbers.

### B73 Accession Numbers

*ZmBBM* (Zm00001eb144510), *ZmWUS2* (Zm00001eb148390), *ZmPLTP* (Zm00001eb406100), *ZmAXIG1* (Zm00001eb271530), *ZmGLB1* (Zm00001eb052450) *Chr.1_ClpP5* (Zm00001eb014750), and *Chr.9_ClpP5* (Zm00001eb396050).

## Results

### Efficiency and Variability of Maize B104 Transformation

**Figure 1:**
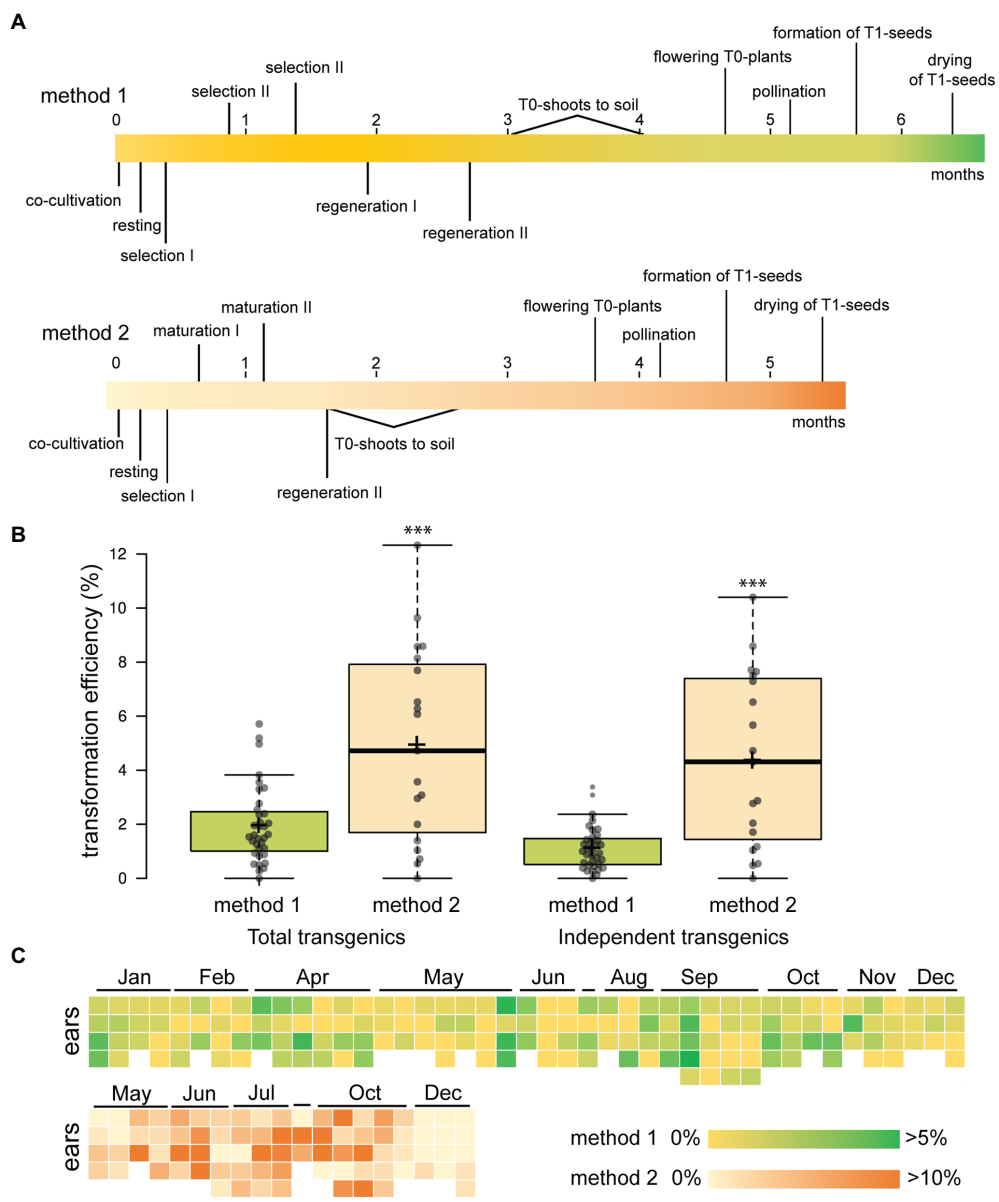
Efficiency and variability of B104 maize transformation. **(A)** Timelines in months of B104 transformation protocols. Key steps are indicated. **(B)** Transformation efficiency of the platform in 2016 using method 1 and in 2020 using method 2. The number of transgenic plants scoring positive for presence of *BIALAPHOS RESISTANCE* (BAR) by lateral flow assays is plotted per starting immature embryo. Efficiency is plotted for both the total number of T0 transgenic plants obtained (left) and independent events (derived from discrete immature embryos, right). *n* = 35 for method 1, *n* = 19 for method 2, ^***^p < 0.001 (Student *t*-test). Center lines show the medians; box limits indicate the 25th and 75th percentiles; whiskers extend 1.5 times the interquartile range from the 25th and 75th percentiles, average is indicated as a cross. **(C)** Heat maps showing transformation efficiency (independent transgenics) per ear ordered chronologically per experiment for 2016 (method 1) and 2020 (method 2). In each experiment, embryos derived from three to five ears were used.

A maize transformation pipeline available at our institute has been used widely by the scientific community for ectopic expression of transgenes (Sun et al., 2017), analysis of reporter genes (Bezrutczyk et al., 2021) and recently, gene editing (Gong et al., 2021; Pedroza-Garcia et al., 2021). We use a well-established method for the public B104 inbred line (Coussens et al., 2012) with minor modifications (method 1). In 2016, we performed 35 comparable experiments according to this protocol and with the same vector backbone and selection marker cassette (pBbm42GW7, Karimi et al., 2013) but with varying transgenes of interest. This allowed us to monitor the efficiency of transformation over several independent experiments (Supplementary Dataset S1). For each transformation experiment, we started with on average 675 immature embryos of 1.5–2 mm as explants (12–14 days after pollination). These were isolated from at least three different ears, harvested from individual greenhouse-grown plants. Embryos were co-cultivated with the hypervirulent *Agrobacterium* strain EHA101, after which callus was induced using the synthetic auxin dicamba and subsequently selected on expression of *BAR* using PPT. After 2.5 months of tissue culture in the dark, embryogenic calli are transferred to a light regime and T0 plantlets are regenerated. After seven months, T1 seeds can be obtained (Figure 1A). On average 12.6 T0 transgenic plants are obtained in each experiment of which at least 7.2 are independent, as they are derived from discrete immature embryos and cannot be clonal. This results in an overall average transformation efficiency of 1.1%, defined as the number of independent T0 plants obtained per starting immature embryo (Figure 1B). There is, however, a large variability between experiments as the efficiency ranged from 0 to 3.4%. Throughout the whole transformation procedure, the embryos derived from different ears were systematically kept separate and transformation efficiencies are counted for each ear (Figure 1C). Within the same experiment, we observed a large variability in the transformation efficiency when comparing results of independent ears, despite being derived from the same inbred line, grown in the same greenhouse, at the same time (Figure 1C). This ear-to-ear variability is known in the field of research (Masters et al., 2020), but rarely reported. With the advent of gene editing, the demand for maize transformation is increasing quickly and solutions are needed that increase transformation efficiencies and cope with the observed variability.

### Enhanced Regeneration and Transformation in B104 Using Modified Tissue Culture Media

A highly efficient maize transformation method using *Agrobacterium* (Lowe et al., 2018) differs from our method 1 on three important points: (a) use of the MRs ZmBBM and ZmWUS2 to boost regeneration (Lowe et al., 2016), (b) use of a ternary vector for *Agrobacterium*, improving T-DNA delivery (Anand et al., 2018), and (c) optimized tissue culture media (Chu et al., 2019).

We first focused on improving tissue culture, without use of MRs. After 14 days of callus induction on dicamba, we introduced a maturation medium inspired by Chu et al. (2019) that holds ABA, cupric sulfate, and the cytokinins zeatin, thidiazuron, and BAP (method 2, Supplementary Tables S4**,**S5). We compared this method with method 1 using embryos derived from the same four ears (Supplementary Table S6). Using method 1, regeneration was poor even in the absence of transformation and selection and only nine out of 87 calli produced shoots. In contrast, method 2 was five weeks faster and shoots were obtained from all 45 starting embryos (Supplementary Table S6).

Next, we applied method 2 for stable transformation, adding PPT as selecting agent (Figure 1A; Supplementary Table S4). During 2020, we performed 19 comparable experiments according to this protocol, with vectors only varying in the transgene of interest or in case of CRISPR/Cas9, sgRNAs (Supplementary Dataset S2). For each construct, we started with on average 322 immature embryos isolated from at least three different ears. This resulted in an average transformation efficiency of 4.2% (independent T0/starting IE, excluding any escapes) compared to 1.1% with method 1 in 2016 (Figure 1B). Again, a large variability was observed between experiments ranging from 0 to 9% and ear-to-ear variability was again observed (Figure 1C). A drawback of this method was the number of additional non-transformed regenerated plants that escaped selection as we found two out of three to be escapes (Supplementary Table S7). In conclusion, changing of the tissue culture procedure alone was sufficient to significantly increase transformation efficiency of B104 and shorten the selection and regeneration time of T0 shoots.

### Use of Morphogenic Genes to Aid B104 Transformation

**Figure 2:**
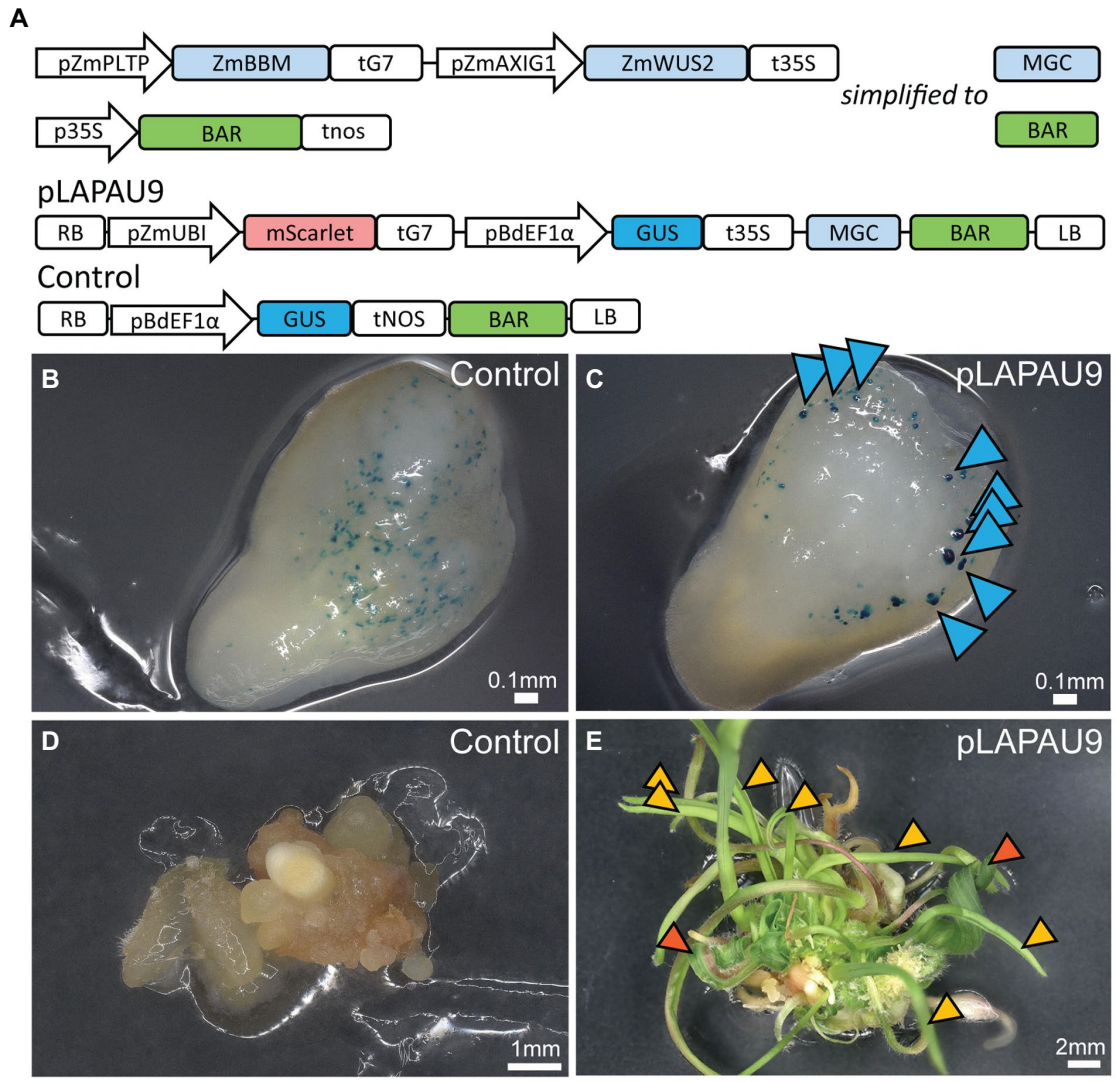
Transformation of B104 using morphogenic genes. **(A)** Diagrams of the T-DNAs used. RB, right border; pZmPLTP, maize PHOSPHOLIPID TRANSFER PROTEIN promoter; pZmAXIG1, *IAA25* auxin-inducible promoter; ZmBBM, maize BABY BOOM; ZmWUS2, maize WUS2, MGC, morphogenic cassette; BAR, BIALAPHOS RESISTANCE; pZmUBI, maize UBIQUITIN-1 promoter; and LB, left border. **(B,C)** B104 immature embryos five days after transformation with control **(B)** or pLAPAU9 **(C)** and stained for GUS expression. Blue arrows indicate somatic proembryos expressing GUS transgene. **(D,E)** Representative images of tissue culture 11 days on maturation II after transformation with control **(D)** or pLAPAU9 **(E)**. Orange arrows indicate wild type appearing shoots, while red arrows indicate abnormal shoots.

Next, we evaluated the combined use of ZmBBM and ZmWUS2 for enhanced plant transformation based on Lowe et al. (2018). We used gene synthesis to create Golden Gate compatible modules for *ZmPLTP* and *ZmAXIG1* promoters and coding sequences of *ZmBBM* and *ZmWUS2*. We then combined these in our standard binary vector with a BAR selection marker, and a GUS and mScarlet-NLS reporter (pLAPAU9, Figure 2A). Immature embryos were transformed with pLAPAU9 and a control construct only containing a *GUS* reporter module and the BAR selection marker (Figure 2A). After five days, control embryos showed a typical pattern of transient *GUS* expression (Figure 2B). Embryos transformed with pLAPAU9 showed larger multicellular protrusions expressing GUS (Figure 2C, blue arrows). These protrusions are likely somatic proembryos as reported earlier (Lowe et al., 2018). Following method 2, the tissue derived from MR-transformed embryos vigorously produced shoots, in contrast to the control (Figures 2D,E). Interestingly, two main shoot types were observed: (a) wild-type shoots that were identified as escapes (Figure 2E, orange arrows) and transgenic shoots with wider, abnormal leaves (Figure 2E, red arrows). In an independent experiment with pLAPAU6 (Supplementary Figure S1A) containing the MRs but no reporters, only five out of 96 tested regenerants were transgenic, and all transgenics had severe developmental defects and did not grow any further (Supplementary Figure S1B). Hence, the MRs also work in B104 to boost direct somatic embryogenesis and regeneration, but our protocol needs further optimization in selection of transgenics and the expression of MRs genes needs tighter control.

### Use of HIGHLY-RESISTANT ACETOLACTATE SYNTHASE as a Selection Marker

**Figure 3:**
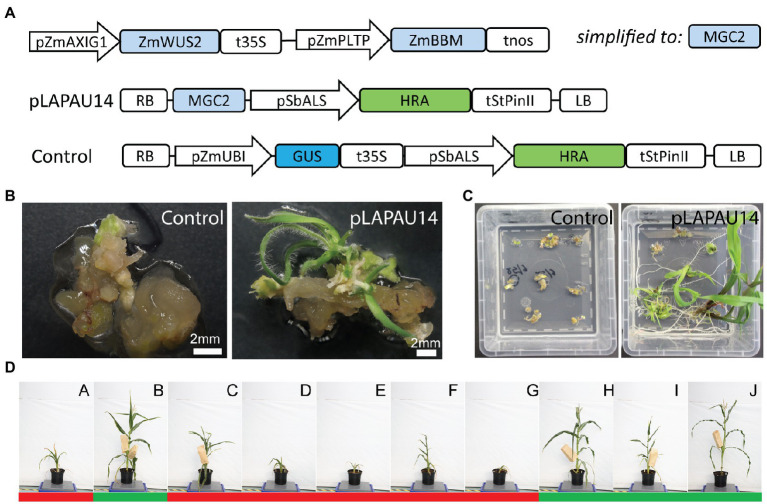
*HIGHLY-RESISTANT ACETOLACTATE* *SYNTHASE (HRA)* as a selection marker for morphogenic regulator-assisted B104 transformation. **(A)** Diagrams of the T-DNAs used. pZmAXIG1, Zm*IAA25* auxin-inducible promoter; pZmPLTP, maize *PHOSPHOLIPID TRANSFER PROTEIN* promoter; ZmBBM, maize *BABY BOOM*; ZmWUS2, maize *WUSCHEL2*, MGC2, morphogenic cassette 2; SbALS, *Sorghum bicolor ACETOLACTATE SYNTHASE* promoter; HRA, *HIGHLY-RESISTANT ACETOLACTATE SYNTHASE*; tStPinII, *Solanum tuberosum PinII* terminator; pZmUBI, maize *UBIQUITIN-1* promoter; RB, right border; and LB, left border. **(B)** Representative images of B104 immature embryos after transformation with pLAPAU14 or a control, 7 days on maturation II media with imazapyr selection. **(C)** Representative images before hardening. **(D)** Images of 10 random T0 plants taken at the same day are shown for pLAPAU14. Letters indicate individual T0 plants. Images were taken 99 days after transfer to soil. Green (fertile) or red (infertile) bars below the plants indicate capacity to produce seeds after backcrossing with wild-type.

It was reported that the use of MRs combined with a shortened time in tissue culture leads to a high number of escapes when using the selection markers BAR and PHOSPHOMANNOSE ISOMERASE (Lowe et al., 2018). It is assumed that selection markers such as *BAR* that deactivate the herbicide also detoxify the regions surrounding the transgenic sectors, allowing escapes. In contrast, selection markers such as HRA (HIGHLY-RESISTANT ACETOLACTATE SYNTHASE; Green et al., 2009) encode enzymes that are insensitive to the selection agents and have been used successfully in combination with MRs (Lowe et al., 2018). Another issue with our selection marker might be the use of the CaMV 35S promoter to drive the *BAR* gene, as it might also influence expression of the neighboring MR-encoding genes, resulting in ectopic expression and unwanted phenotypes later in development (Jopcik et al., 2014). Hence, based on Lowe et al. (2018), we cloned *HRA* driven by the *Sorghum bicolor ACETOLACTATE SYNTHASE* (*SbALS*) promoter as a selection marker (Figure 3A). In addition, we changed the order of the morphogenic genes in the T-DNA, resembling earlier reports more closely (Figure 3A; Lowe et al., 2018).

Transformation of a pZmUBI::*GUS* control construct with the pSbALS::*HRA* selection marker yielded no transformants from 212 starting embryos derived from four ears (Supplementary Table S8). However, when we combined pSbALS::*HRA* with the MRs on the same T-DNA (vector pLAPAU14), independent transgenics were obtained at a transformation efficiency of 6.1% without any escapes as determined by PCR (Supplementary Table S8). While plants initially appeared wild-type (Figures 3B–D), several transformed plants again showed developmental defects after hardening and only four out of 10 were able to produce seed (Figure 3D). We confirm that the selection marker HRA is well-suited to perform rapid selection in combination with MRs, and produces no escapes, while maintaining a transformation efficiency similar to method 2 (~4%) using *BAR*. However, replacement of the selection marker module was not sufficient to abolish high or ectopic expression of MRs later in development.

### Gene Excision Reduces T-DNA Copy Number and Yields Fertile Plants

**Figure 4:**
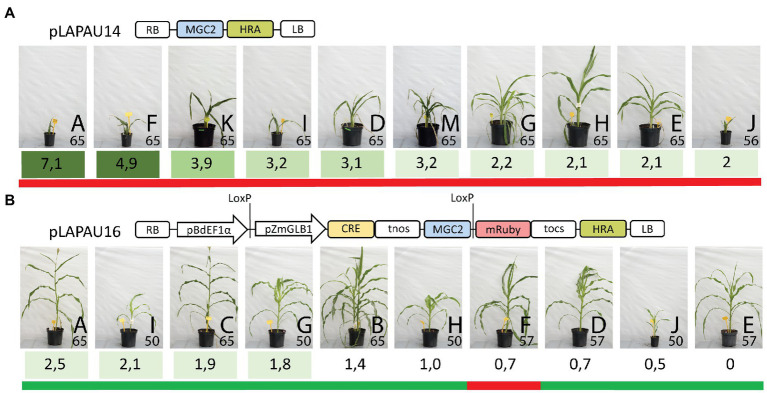
Relation between morphogenic regulator copy number and overall plant growth and fertility. Images of 10 random T0 plants taken at the same day are shown for pLAPAU14 **(A)** and pLAPAU16 **(B)**. The T-DNA structure is shown schematically as in Figure 3, except HRA represents pSbALS::HRA:tStPinII. Letters indicate individual T0 plants and are ranked from left to right according to their estimated pZmPLTP::*ZmBBM* copy number (shaded green). The number in the image indicates the days after transfer to soil the pictures were taken. Green (fertile) or red (infertile) bars below the plants indicate capacity to produce seeds after backcrossing with wild-type.

To reduce the effect of the MRs later in development, we cloned a Cre/LoxP recombination system similar to Hoerster et al. (2020), in which a monocot codon-optimized Cre recombinase (MoCre) is driven by the promoter of the late-embryogenesis and ABA-inducible maize *GLOBULIN-1* gene (pZmGLB1; Duncan et al., 2003). LoxP sites flank the MRs and the pZmGLB1::*MoCre* module (pLAPAU16; Figure 4). Upon induction of MoCre after somatic embryo formation and/or induction by ABA in tissue culture media (Supplementary Table S4), LoxP sites are recombined, potentially removing the developmental regulators from the genome and thus eliminating the detrimental ectopic expression of *ZmBBM* and *ZmWUS2* (Figure 4; Hoerster et al., 2020).

Similar as above, transformation of the pZmUBI::*GUS* control with HRA selection marker only yielded two transformants from 216 starting embryos derived from four ears (0.9%, Supplementary Table S8). Using embryos derived from the same four ears, pLAPAU14 (without excision) yielded 15.3% independent transgenics and pLAPAU16 (with excision) 5.1% (Supplementary Table S8). For each construct, we continued with ten random independent transformants. While all ten plants transformed with pLAPAU14 again showed developmental defects and were infertile (Figure 4A), plants transformed with pLAPAU16 appeared wild-type and all but one were fertile (Figure 4B; after crossing to a WT B104 plants, seeds were produced).

To investigate this phenotypic difference between pLAPAU14 and pLAPAU16 transgenic plants in detail, we used ddPCR to examine T-DNA copy number in leaf samples of the ten plants generated with pLAPAU14 and pLAPAU16 (Supplementary Table S9). Primers targeting the pZmPLTP::*ZmBBM* heterologous fusion or the *StPinII HRA* terminator were used to amplify only T-DNA encoded genes. pZmPLTP::*ZmBBM* was present in on average 3.3 copies per T0 plant with pLAPAU14, while with pLAPAU16 the average was 1.3 copies (Supplementary Table S9). Interestingly, the copy number of the *HRA* resistance marker outside the LoxP sites was reduced from on average 2.9 copies for pLAPAU14 to 1.6 for pLAPAU16. Moreover, while two out of ten plants transformed with pLAPAU14 did not contain the *aadA* bacterial selection marker gene from the vector backbone, six out of ten transformed with pLAPAU16 lacked the *aadA* gene. This suggests that Cre-mediated recombination between LoxP sites may also reduce T-DNA complexity by excising T-DNA tandem repeats (De Buck et al., 2007). Finally, the observed non-integer ddPCR values (e.g., 0.5) for pZmPLTP::*ZmBBM* as compared to *HRA*, and specifically in plants transformed with pLAPAU16 compared to pLAPAU14 suggest that pLAPAU16-transformed plants are genetic mosaic for excision events. Only one out of ten plants transformed with pLAPAU16 (Plant E) was identified with complete loss of morphogenic genes in T0 leaf material.

In summary, combining MRs with a Cre/LoxP-mediated excision system maintained their functionality and resulted in a high transformation efficiency. Use of the Cre/LoxP-system was associated with a reduced MR copy number, a less complex T-DNA structure, and normal plant development and fertility. Nevertheless, obtained plants in most cases still contained the MR cassettes.

### Gene Editing in Maize B104 Using *VYL* as a Marker Gene

To evaluate gene editing in maize, we aimed to target a gene that leads to a visible knockout phenotype, but without severe developmental and fertility effects. Markers that have been used before such as *Zmzb7* lead to albino phenotypes, and are therefore lethal (Feng et al., 2016). The gene *VYL* (*Chr.9_ClpP5*) was identified in an ethyl methane sulfonate-mutagenized population derived from the maize inbred line B73 (Xing et al., 2014a). B73vyl homozygous mutant maize plants exhibit a yellow leaf phenotype after emergence but gradually recover and become indistinguishable from wild-type plants after approximately 2 weeks. *B73vyl* (Zm00007a00050679 in B104) encodes for Chr.9_ClpP5, a proteolytic subunit of the chloroplast Clp protease complex. The B73*vyl* mutation was found to be a 141 bp insertion in the fourth exon that likely results in a knockout (Xing et al., 2014a). The B73 genome contains a duplicated paralog *Chr.1_ClpP5* (Zm00007a00035036 in B104) that explains the lack of more severe phenotypes.

**Figure 5:**
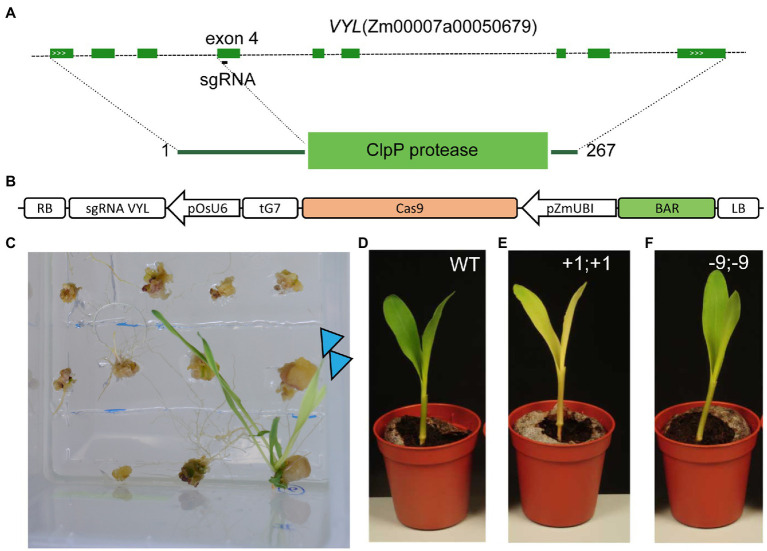
Loss of *VIRESCENT YELLOW-LIKE* (VYL) function in B104 results in a pale-yellow phenotype. **(A)** Genomic structure of the B104 *VYL* *(Chr.9_ClpP5)* gene and location of the sgRNA. Dark green boxes designate exons; light green boxes, UTRs; solid lines, introns; and white arrows gene orientation. **(B)** Diagram of the T-DNA used for CRISPR/Cas9 gene editing in maize. LB, left border; BAR, *BIALAPHOS RESISTANCE*, pZmUBI, *maize UBIQUITIN-1* promoter; and RB, right border. **(C)** Loss-of-function phenotype of T0 regenerants during the final tissue culture step of maize transformation. Edited T0 plants are indicated with an arrow. **(D–F)** Seven-day-old seedling phenotype of wild-type, knock-out (+1;+1, homozygous 1 bp insertion) and a weak allele (−9;−9, homozygous 9 bp deletion).

To mimic the B73vyl mutant, we designed a CRISPR/Cas9 construct with a sgRNA targeting exon 4 of *VYL* (Figures 5A,B). The plasmid was transformed in B104 zygotic embryos using method 1. Already during the regeneration of the plantlets in tissue culture, the expected pale-yellow phenotype was observed in transgenic plants (Figure 5C). Eleven T0 plants, of which five were independent and derived from different zygotic embryos, were analyzed for the *VYL* locus. Similar to earlier reports with gene editing in B104 (Char et al., 2017), CRISPR/Cas9 gene editing was very efficient with all but one transgenic plant having indel scores of >80% based on Sanger sequencing and subsequent estimation of the efficiency of mutations (Supplementary Table S10). At least three plants had three or more detected alleles, indicating these T0 plants were genetic mosaic, others were homozygous or transheterozygous for edits (Supplementary Table S10). Similar to B73vyl, T0 mutant lines recovered phenotypically as adults, were fertile and edits could be inherited after self-crossing. In T2, we obtained Cas9 null-segregants with homozygous mutations causing a frameshift to show a pale-yellow phenotype compared to WT (Figures 5D,E). Interestingly, we also isolated a weak allele with a three amino acid deletion resulting in an intermediary phenotype (Figure 5F). In conclusion, CRISPR/Cas9 gene editing is efficient and heritable in B104 and *VYL* is a convenient marker that can be targeted for gene editing in B104.

### Use of MRs in Combination With CRISPR/Cas9

**Figure 6:**
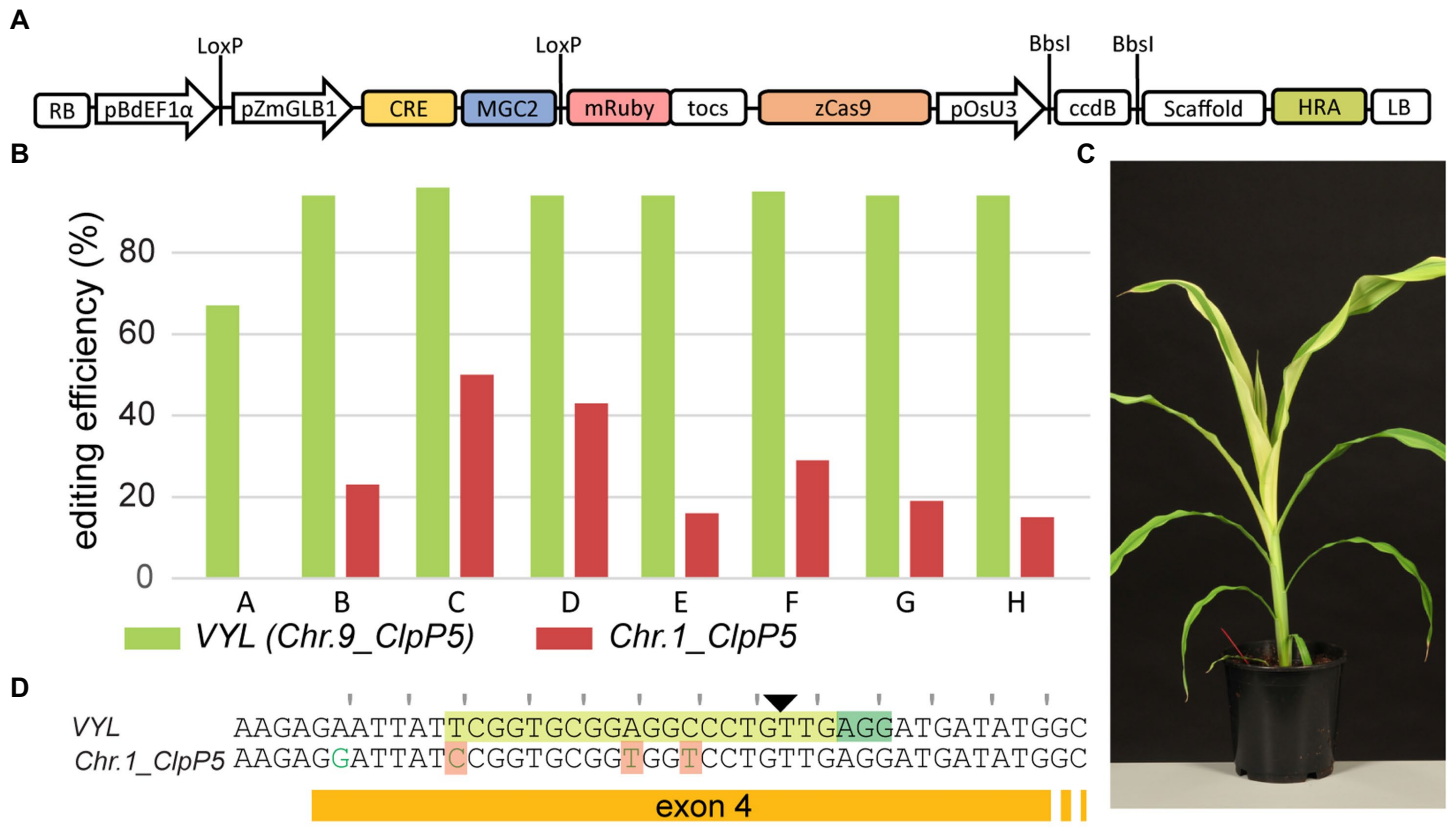
Use of morphogenic regulators for efficient gene editing in B104. **(A)** The pLAPAU17 construct allowing direct cloning of a spacer sequence. The T-DNA structure is shown schematically as in Figure 4. **(B)** For eight independent T0 lines, the editing efficiency is plotted at *VYL* (*Chr.9_ClpP5*; green bars) and *Chr.1_ClpP5* (red bars). **(C)** Representative image of a T0 plant (line B) showing albino mosaicism. **(D)** The targeted B104 genomic sequences of *VYL* and *Chr.1_ClpP5*. PAM is highlighted in dark green, spacer in light green, and the Cas9 cut site is indicated with a triangle. Mismatched bases in the spacer are highlighted in orange. The reading frame is marked.

Continued presence of the MR cassette is undesirable for the generation of stable transgenic lines and downstream phenotypic analysis. However, for gene editing applications, MRs linked to a CRISPR module on a single T-DNA can be removed in the next generation after a genetic cross, creating edited null-segregants. Hence, we constructed the vector pLAPAU17 that contains outside the LoxP recombination sites an additional module with a maize codon-optimized Cas9 variant (Xing et al., 2014b) driven by a *ZmUBI* promoter and a pOsU3 module that allows direct cloning of the spacer (Figure 6A). We then introduced the same *VYL* targeting spacer used before (Figure 5) and transformed 222 immature maize embryos. In total, 17 transgenic plants were obtained of which at least nine were independent (4% transformation efficiency). T0 plants were edited in *VYL* with high efficiency (Figure 6B, green bars; Supplementary Table S10). Some T0 plants unexpectedly showed phenotypic mosaicism with albino stripes, not seen in previous experiments targeting *VYL* (Figure 6C). The spacer used for *VYL* (*Chr.9_ClpP5*) contains three mismatches to the *Chr.1_ClpP5* sequence, making the latter a potential off-target (Figure 6D). Indeed, T0 plants also showed editing at the corresponding site in *Chr.1_ClpP5* (Figure 6B, red bars; Supplementary Table S10). Remarkably, none of the eleven lines generated with the previous CRISPR/Cas9 construct targeting *VYL* (Figure 5) showed editing at *Chr.1_ClpP5* (Supplementary Table S10), suggesting that one or more factors in the pLAPAU17 construct make CRISPR/Cas9 editing more active at the off-target *Chr.1_ClpP5* locus. Line B, generated with pLAPAU17-*VYL*, scoring −4;+1 for *VYL* and 0;+1 (23%) for *Chr.1_ClpP5*, was selected and backcrossed to WT B104. The T-DNA segregated in a Mendelian fashion for a single T-DNA locus and T1 null-segregants were identified using PCR. Confirming inheritance of edits, T1 null-segregants were all heterozygous for a mutated *VYL* allele, and either WT or heterozygous for a mutated *Chr.1_ClpP5* allele (Supplementary Table S10). In conclusion, MRs can be readily combined with CRISPR/Cas9 for efficient transformation and heritable gene editing in B104 maize.

## Discussion

In this work, we have described methods to significantly improve B104 transformation with and without the use of the MR genes *BBM* and *WUS*. The methods reduce the time between co-cultivation with *Agrobacterium* and the generation of a T0 plant, thereby also reducing the workload for the researchers performing tissue culture. Our results show that a successful translation of the BBM-WUS technology to the B104 inbred in an academic setting requires careful consideration and evaluation of other transformation parameters, including tissue culture media and selection markers.

The tissue culture methods 1 and 2 investigated in this work include the growth regulator dicamba instead of 2,4-D from co-cultivation up until the first selection step. Dicamba promotes the formation of embryogenic callus from immature zygotic embryos in tropical maize (Akoyi et al., 2013). In B104, we also observed improved embryogenic callus formation, and dicamba now replaces 2,4-D in the tissue culture procedures of our B104 maize transformation platform. In addition, we introduced in method 2 the use of a maturation medium with cytokinins (6-BAP, zeatin, and TDZ) and CuSO_4_, accelerating the regeneration time and increasing the efficiency of transformation. Nevertheless, use of method 2 with BAR as a selection marker resulted in numerous escapes prompting further improvement of the method by evaluation of other selection agents and/or regimes.

Only a single ear per plant is pollinated and used for embryo production. By systematically keeping track of which ear the immature embryos are derived from, we were able to provide a unique report on the ear-to-ear variability of maize transformation. Despite the use of inbred lines, grown side-by-side in a controlled greenhouse environment, different ears yield different transformation efficiencies, irrespective of season, year, or transformation method used. When using MRs, ear-to-ear variability has also been observed (Lowe et al., 2018; Masters et al., 2020). The reason for this variability might be related to slight physiological differences between the individual plants that might be caused by position effects in the greenhouse resulting in slight variability in illumination and temperature. Alternatively, minor insect infestation of the plants could lead to a suboptimal competence for *Agrobacterium* infection of the harvested zygotic embryos of a particular ear. In order to obtain more standardized transformation efficiencies, we correct for this ear-to-ear variability by using embryos from at least three ears per construct. This has practical implications as despite rising transformation efficiencies, the starting number of plants for embryo production will likely need to remain the same per construct, but with embryos from one plant distributed over multiple constructs. Improvements in the use of other explants such as seedling-derived leaf tissue might be needed to eventually enable maize transformation outside dedicated facilities (Kausch et al., 2021).

There are several additional parameters that we did not evaluate in this study and could further improve B104 transformation using MRs. For example, our study was limited to the *Agrobacterium* strains EHA101 and EHA105, typically used for B104 transformation (Frame et al., 2006). Previous reports on use of MRs used the auxotrophic *Agrobacterium* strain LBA4404 Thy^-^ (Lowe et al., 2018; Masters et al., 2020). Auxotrophic strains allow lowering of antibiotics concentrations in tissue culture media to remove Agrobacteria in the later stages of plant transformation. A reduced use of antibiotics may positively impact plant regeneration in addition to ensuring biocontainment of engineered strains. Recently, auxotrophic EHA101 and EHA105 strains have been developed for academic use (Aliu et al., 2020) or can be easily constructed using base editing methods adapted for *Agrobacterium* (Rodrigues et al., 2021). The *Agrobacterium* strain used for transformation may also influence T-DNA copy number (Cho et al., 2014; Zhi et al., 2015), which we show here to be pivotal for successful use of MRs. Previous reports on the use of pZmPLTP::*ZmBBM* pZmAXIG1::*ZmWUS2* combined the MRs with ternary vectors to increase susceptibility to *Agrobacterium* (Lowe et al., 2018; Masters et al., 2020). Ternary vectors equip Agrobacteria with extra virulence (*vir*) genes from TiBo542 (Anand et al., 2018; Zhang et al., 2019a). In this study, we examined the use of MRs independent of ternary vectors. Ternary vectors have now been developed and made available for academic use (Zhang et al., 2019a) and it will be interesting to examine their combination with pZmPLTP::*ZmBBM* pZmAXIG1::*ZmWUS2* in transformation of B104.

Despite the use of the pZmPLTP and pZmAXIG1 promoters to drive MRs, we report that T0 plants with high MR copy numbers have severe developmental defects and cannot produce seeds, which make these plants unsuitable for applications such as gene editing. In Lowe et al. (2018), only plants containing a single T-DNA copy were grown in the greenhouse, probably masking this effect. Nevertheless, multicopy events with abnormalities derived from particle bombardment were also reported (Lowe et al., 2018). The pleiotropic effects of continued presence of MRs are likely also genotype-dependent. In *Sorghum bicolor*, single copy T0 plants of one genotype did not show severe abnormalities, while two other genotypes showed reduced fertility (Che et al., 2021). As events with a high copy number of MRs are infertile in B104, these plants cannot be used for most gene editing applications that require outcrossing of the T-DNA. Hence, for successful use of MRs for gene editing, low-copy events need to be preselected using techniques such as digital PCR. It is well-known that T-DNA loci often contain multiple T-DNA copies, often in complex arrangements including direct and inverted repeats, and also may contain vector backbone (Jupe et al., 2019). This was also observed here in our experimental setup. While multiple copies may result in higher transgene expression in T0, multi-copy inverted repeats have been associated with transgene silencing in later generations (De Buck et al., 2007). This instability results in single-copy events being desired in industry (Que et al., 2014). A solution also explored here to reduce MR copy number is to flank MRs with LoxP sites and use a developmentally-triggered Cre recombinase to excise the MRs (Masters et al., 2020). Interestingly, we observed a marked reduction in copy number of the entire T-DNA, and a reduction in backbone integration. This can be explained by excision of direct T-DNA repeats (De Buck et al., 2007). Alternative approaches have been reported to exclude MR expression late in development. These approaches rely on transient expression of MRs without integration in combination with selection of the T-DNA of interest (Hoerster et al., 2020; Aregawi et al., 2021; Nelson-Vasilchik et al., 2022).

We observed that T0 plants showing evidence of Cre/LoxP-mediated excision also still contained MRs. This suggests that they are genetic mosaic for excision events, likely by the activation of the *GLB1* promoter driving *Cre* late during somatic embryogenesis. Nevertheless, because of the fertility of the resulting T0 plants, they can be used. Especially in applications for which gene editing efficiency is low such as homologous recombination (Barone et al., 2020), a high number of transformants is beneficial. Here, we targeted *VYL* (*Chr.9_ClpP5*) using CRISPR/Cas9 for loss-of-function. *VYL* is a promising marker gene as it results in a visual phenotype in tissue culture, without affecting further plant growth. Such visual markers are especially interesting in co-editing approaches, targeting a gene of interest together with a visual marker using CRISPR/Cas9 multiplexing (Zhang et al., 2019b). Alternatively, (co-)editing *VYL* can be used in methods that do not allow use of genetically encoded selection markers such as gene editing using bombardment with CRISPR ribonucleoproteins (Dong et al., 2021). An unexpected observation in this work is the off-target editing of *Chr.1_ClpP5* with the pLAPAU17-*VYL* construct. Further research is needed if this increased editing efficiency at this off-target locus can be attributed to the use of MRs, the different Cas9 variant used or the promoter driving the sgRNA. For future co-editing applications, *VYL* spacers can be designed that are more specific for *VYL*, while the spacer used here can be useful for future off-target research in maize. An off-target analysis in maize suggested to have at least one mismatch in the seed region combined with at least three additional mismatches in the PAM distal region (Young et al., 2019). The targeted region in *VYL* had three mismatches with the *Chr.1_ClpP5* site, of which one in the PAM proximal seed region. Our data show that even three mismatches is not enough to prevent off-target activity at some loci and careful sgRNA design needs to be combined with genotyping of predictable off-targets when possible.

Interestingly, pLAPAU17-*VYL* T0 plants were genetic mosaic for editing at *Chr.1_ClpP5*, which correlated with the phenotypic albino mosaicism also observed in these plants. Previously, *Chr1._ClpP5* was identified as a modifier locus of the B73*vyl* loss-of-function mutation. Combination of B73*vyl* with *Chr.1_ClpP5* alleles of different inbreds led to phenotypes of varying severity. This was explained by functional redundancy of the two encoded proteins, combined with *cis*-regulatory variation between the *Chr.1_ClpP5* alleles (Xing et al., 2014a). In *Arabidopsis*, ClpP5 is encoded by a single gene and loss-of-function mutants appear embryo lethal (Kim et al., 2009). Whether or not embryo lethality is also the case for maize can be investigated with the lines generated in this research.

The GROWTH REGULATORY FACTOR (GRF)/GRF INTERACTING FACTOR (GIF) signaling pathway recently emerged as an alternative to BBM-WUS to improve regeneration in monocots (Debernardi et al., 2020; Kong et al., 2020). It was serendipitously found that expression of a TaGRF4-TaGIF1 chimeric protein under control of pZmUBI enhances regeneration in wheat and resulted in a doubling in transformation efficiency for the wheat variety Fielder and allowed transformation of otherwise recalcitrant varieties (Debernardi et al., 2020). An important benefit of this technology is that adult plants have a wild-type phenotype, likely by miRNA396-regulation of the chimera (Debernardi et al., 2020). In maize, expression of TaGRF1 orthologs under control of pBdEF1a also improved transformation (Kong et al., 2020). We expect that our experience with BBM-WUS shared in this study will also help translating GRF-GIF and upcoming MR-based technologies to maize.

## Data Availability Statement

The raw data supporting the conclusions of this article will be made available by the authors, without undue reservation.

## Author Contributions

LP designed the research. SA, LI, GC, EL, RV, YV, SB, and AW performed maize transformation experiments and data analysis. MK designed constructs and performed cloning. LD and EK performed ddPCR experiments and data analysis. LP, ML, TJ, and DI supervised the research. LI and LP wrote the manuscript. All authors contributed to the article and approved the submitted version.

## Funding

This work was supported by Ghent University Bijzonder Onderzoeksfonds Methusalem Project BOF.MET.2015.0002.01.

## Conflict of Interest

The authors declare that the research was conducted in the absence of any commercial or financial relationships that could be construed as a potential conflict of interest.

## Publisher’s Note

All claims expressed in this article are solely those of the authors and do not necessarily represent those of their affiliated organizations, or those of the publisher, the editors and the reviewers. Any product that may be evaluated in this article, or claim that may be made by its manufacturer, is not guaranteed or endorsed by the publisher.

## Footnotes

^1^ http://crispor.tefor.net/

## Supplementary Material

Supplementary Figure S1 Expression of morphogenic regulators causes developmental defects.

Supplementary Dataset S1 Transformation results using method 1.

Supplementary Dataset S2 Transformation results using method 2.
