# Supplementary material for: Optimized Transformation and Gene Editing of the B104 Public Maize Inbred by Improved Tissue Culture and Use of Morphogenic Regulators
Source: Front Plant Sci. 2022 Apr 22;13:883847. doi: 10.3389/fpls.2022.883847 (PMC9072829; doi:10.3389/fpls.2022.883847)
Supplement: Supplementary file 1 [file Data_Sheet_1.PDF]

Table S1 Plasmids used in this study**.** StR, streptomycin resistance; KmR, kanamycin resistance; SpR, spectinomycin resistance; AmpR, ampicillin resistance.

**Name**	**Features**	**Source**
pBbm42GW7	Destination vector, SpR	Karimi *et al*., 2013
pMR185	Shuttle cloning vector for sgRNA, KmR	Thripati *et al*, 2019
pGG-A-C	Empty Golden Gate entry vectors, AmpR	Rodrigues *et al*., 2021
pRA-AG	Green Gate destination vector, SpR	This work
pRA-U1-AG-U9	Gibson destination vector, SpR	This work
pGG-A000, pGG-C000	Empty Golden Gate entry vectors, AmpR	Lampropoulos et al., 2013
pPZP200	Source of backbone	Hajdukiewicz *et al*., 1994
pEN-L4-UBIL-R1	Template for ZmUBI	Karimi *et al*., 2007
pEN-L4-pZmUBI-Cas9PTA-G7-R1	MultiSite Gateway entry clone, KmR	This work
pEN-L4-AG-R1	Empty MultiSite Gateway entry clones, KmR	Houbaert *et al*., 2018
pEN-L4- pZmUBI-mScarlet-NLS-G7T-R1	MultiSite Gateway entry clone, KmR	This work
pEN-R2-pBdEF1a-GUS-35ST	MultiSite Gateway entry clone, KmR	This work
pEN-L4-pZmPLTP-ZmBBM-G7T-R1	MultiSite Gateway entry clone, KmR	This work
pEN-L1-pZmAXIG1-ZmWUS2-35ST-L2	MultiSite Gateway entry clone, KmR	This work
pGG-A-pZmUBI-B	ZmUBI promoter, AmpR	gatewayvectors.vib.be
pGG-A-pZmUBI.2-B	ZmUBI promoter, original splice acceptor site, AmpR	gatewayvectors.vib.be
pGG-A-pBdEF1a-B	BdEF1a promoter, start codon, AmpR	gatewayvectors.vib.be
pGG-A-pBdEF1a.2-B	BdEF1a promoter, no start codon, AmpR	This work
pGG-A-pZmPLTP-C	Scutellum epithelium specific promoter, AmpR	This work
pGG-A-pZmAXIG1-C	Auxin-inducible promoter, AmpR	This work
pGG-A-pZmGLB1-C	Late-embryogenesis promoter, AmpR	This work
pGG-A-pZmUBI-mScarlet-NLS-G7T-pBdEF1a-GUS-35ST-B	Green Gate Entry clone, AmpR	This work
pGGW-A-m43GW-B	Golden Gateway vector	Karimi and Jacobs, 2020
pGG-B-pZmPLTP-ZmBBM-G7T-pZmAXIG1-ZmWUS2-35ST-C	Green Gate Entry clone, AmpR	This work
pGGW-B-m43GW-C	Golden Gateway vector	Karimi and Jacobs, 2020
pGG-C-ZmBBM-D	Morphogenic regulator, AmpR	This work
pGG-C-ZmWUS2-D	Morphogenic regulator, AmpR	This work
pGG-C-MoCRE-D	CRE recombinase (monocot codon optimized), AmpR	This work
pGG-mScarlet-NLS-D	Fluorescent marker, AmpR	This work
pGG-C-GUS-D	GUS marker	Lampropoulos et al., 2013
pGG-C-Cas9PTA*- D	Arabidopsis codon optimized Cas9	gatewayvectors.vib.be
pGG-C-zCas9-D	Maize codon optimized Cas9	gatewayvectors.vib.be
pGG-C-mRUBY-ATG-D	mRUBY without start codon	This work
pGG-E-tG7-F	tG7 terminator, AmpR	gatewayvectors.vib.be

pGG-D-tocs-G	Terminator, AmpR	gatewayvectors.vib.be
pGG-D-tG7-G	Terminator, AmpR	gatewayvectors.vib.be
pGG-tnos-G	Terminator, AmpR	gatewayvectors.vib.be
pGG-D-t35S-G	Terminator, AmpR	gatewayvectors.vib.be
pEN-R2-linker-L3	Linker	gatewayvectors.vib.be
pEN-L1-linker-L2	Linker	gatewayvectors.vib.be
pGG-B-linker-C, pGG-C-linker-G	Linker, AmpR	Lampropoulos et al., 2013
pGG-D-linker-E	Linker, AmpR	gatewayvectors.vib.be
pGGIB-U1-linker-U3	Gibson linker, KmR	This work
pGGIB-U4-linker-U6	Gibson linker, KmR	This work
pGGIB-U7-linker-U9	Gibson linker, KmR	This work
PGGIB-U6-linker-U9	Gibson linker, KmR	This work
pGGIB-U8-linker-U9	Gibson linker, KmR	This work
pGGIB-U1-pBdEF1a-LoxP-G7t-U2	Gibson entry vector, KmR	This work
pGGIB-U2-pZmGLB1-MoCRE-tnos-U3	Gibson entry vector, KmR	This work
pGGIB-U4-pZmPLTP-ZmBBM-tnos-U5	Gibson entry vector, KmR	This work
pGGIB-U5-LoxP-mRUBY3-tocs	Gibson entry vector, KmR	This work
pGGIB-U6-pZmUBI-zCas9-tG7-U7	Gibson entry vector, KmR	This work
pGGIB-U7-pOsU3-BsaI-ccdB/GmR-BsaI-U8	Gibson entry vector, KmR	This work
pBbm42GW7-pOsU6-sgRNA(VYL)-pZmUBIL-Cas9PTA-G7t	Expression vector, SpR	This work
pLAPAU6	Expression vector, SpR	This work
pLAPAU9	Expression vector, SpR	This work
pRA-ZmUBI-GUS-35ST	Expression vector, SpR	This work
pLAPAU14	Expression vector, SpR	This work
pLAPAU16	Expression vector, SpR	This work
pLAPAU17	CRISPR/Cas9 destination vector, SpR	This work

Table S2. Oligonucleotides used in this study

Name	Sequence (5'-3')	Description
**sgRNA cloning**		
CROPGEN7	GTTGTCGGTGCGGAGGCCCTGTTG	*VYL spacer*
CROPGEN8	AAACCAACAGGGCCTCCGCACCGA	*VYL spacer*
CROPGEN27	GGCATCGGTGCGGAGGCCCTGTTG	*VYL spacer*
**Genotyping**		
CROPGEN6	CCATTCCTAACAGGAAACGAA	*VYL (Chr.9_ClpP5)*
CROPGEN56	GGGCTTGTTTGGTTGGATACT	*VYL (Chr.9_ClpP5)*
CROPGEN452	TAGAGTGAATATGACTGCAGAGG	*Chr.1_ClpP5*
CROPGEN453	GATTTGGTCGATCAGGTGGAAT	*Chr.1_ClpP5*
CROPGEN597	GATCCGAATTGAGAACCTCCCA	*HRA*
CROPGEN598	TGAAATGCATCTGGTTCATCAAAGA	*StPinII terminator*
**Cloning**		
UBI_Fw	TTTTGGTCTCAACCTGTGCAGCGTGACCCGGTCGTGC	A-ZmUBI-B cloning
UBI_Rv	TTTTGGTCTCATGTTTGCAGAAGTAACACCAAACAAC	A-ZmUBI-B cloning
B1_A-LOX_F	ACCTTCAGCCTGATCCTGAGCTTCTAGCATAACTTCGTATAGCATACATTATACGAAGTTAT	Cloning A-LoxP-C
B1_C-LOX_R	AGCCATAACTTCGTATAATGTATGCTATACGAAGTTATGCTAGAAGCTCAGGATCAGGCTGA	Cloning A-LoxP-C
CROPGEN493	TGTggtctcaggctccatgTTACGTCCTGTAGAAAC	C-GUS intron-D cloning
CROPGEN494	TGTggtctcactgatcaTTGTTTGCCTCCCTGCTG	C-GUS intron-D cloning
**ddPCR**		
CROPGEN710	atctcgttggggatgtcttg	*FPGS* (reference)
CROPGEN711	agcaccgttcaaatgtctcc	*FPGS* (reference)
CROPGEN773	gaatgtgtgttgggtttgcat	*ADH1* (reference)
CROPGEN774	tccagcaatccttgcacctt	*ADH1* (reference)
CROPGEN638	cacacaactttgatgcccacat	PinIIt (HRA terminator)
CROPGEN639	gacttgtccatcttctggattgg	PinIIt (HRA terminator)
CROPGEN632	aaagcgagccagttgttcac	pPLTP::BBM (transgene)
CROPGEN633	cattccaaacagcccgacga	pPLTP::BBM (transgene)
CROPGEN672	ttctccgcgctgtagaagtc	*aadA* (backbone)
CROPGEN673	ccaaattgcagttcgcgctt	*aadA* (backbone)

Table S3 DNA sequences of elements synthesized in this study

Bsa1-C-ZmBBM-D-Bsa1 (2167bp)

ggtctcaggctccatggccactgtgaacaactggctcgctttctccctctccccgcaggagctgccgc
cctcccagacgacggactccacactcatctcggccgccaccgccgaccatgtctccggcgatgtctgc
ttcaacatcccccaagattggagcatgaggggatcagagctttcggcgctcgtcgcggagccgaagct
ggaggacttcctcggcggcatctccttctccgagcagcatcacaaggccaactgcaacatgataccca
gcactagcagcacagtttgctacgcgagctcaggtgctagcaccggctaccatcaccagctgtaccac
cagcccaccagctcagcgctccacttcgcggactccgtaatggtggcctcctcggccggtgtccacga
cggcggtgccatgctcagcgcggccgccgctaacggtgtcgctggcgctgccagtgccaacggcggcg
gcatcgggctgtccatgattaagaactggctgcggagccaaccggcgcccatgcagccgagggtggcg
gcggctgagggcgcgcaggggctctctttgtccatgaacatggcggggacgacccaaggcgctgctgg
catgccacttctcgctggagagcgcgcacgggcgcccgagagtgtatcgacgtcagcacagggtggag
ccgtcgtcgtcacggcgccgaaggaggatagcggtggcagcggtgttgccggcgctctagtagccgtg
agcacggacacgggtggcagcggcggcgcgtcggctgacaacacggcaaggaagacggtggacacgtt
cgggcagcgcacgtcgatttaccgtggcgtgacaaggcatagatggactgggagatatgaggcacatc
tttgggataacagttgcagaagggaagggcaaactcgtaagggtcgtcaagtctatttaggtggctat
gataaagaggagaaagctgctagggcttatgatcttgctgctctgaagtactggggtgccacaacaac
aacaaattttccagtgagtaactacgaaaaggagctcgaggacatgaagcacatgacaaggcaggagt
ttgtagcgtctctgagaaggaagagcagtggtttctccagaggtgcatccatttacaggggagtgact
aggcatcaccaacatggaagatggcaagcacggattggacgagttgcagggaacaaggatctttactt
gggcaccttcagcacccaggaggaggcagcggaggcgtacgacatcgcggcgatcaagttccgcggcc
tcaacgccgtcaccaacttcgacatgagccgctacgacgtgaagagcatcctggacagcagcgccctc
cccatcggcagcgccgccaagcgcctcaaggaggccgaggccgcagcgtccgcgcagcaccaccacgc
cggcgtggtgagctacgacgtcggccgcatcgcctcgcagctcggcgacggcggagccctggcggcgg
cgtacggcgcgcactaccacggcgccgcctggccgaccatcgcgttccagccgggcgccgccagcaca
ggcctgtaccacccgtacgcgcagcagccaatgcgcggcggcgggtggtgcaagcaggagcaggacca
cgcggtgatcgcggccgcgcacagcctgcaggacctccaccacctgaacctgggcgcggccggcgcgc
acgacttttttctcggcagggcagcaggccgccgccgctgcgatgcacggcctgggtagcatcgacagt
gcgtcgctcgagcacagcaccggctccaactccgtcgtctacaacggcggggtcggcgacagcaacgg
cgccagcgccgtcggcggcagtggcggtggctacatgatgccgatgagcgctgccggagcaaccacta
catcggcaatggtgagccacgagcaggtgcatgcacgggcctacgacgaagccaagcaggctgctcag
atggggtacgagagctacctggtgaacgcggagaacaatggtggcggaaggatgtctgcatgggggac
tgtcgtgtctgcagccgcggcggcagcagcaagcagcaacgacaacatggccgccgacgtcggccatg
gcggcgcgcagctcttcagtgtctggaacgacacttaatcagtgagacc

Bsa1-C-ZmWUS2-D-Bsa1(937bp)

ggtctcaggctccatggcggccaatgcgggcggcggtggagcgggaggaggcagcggcagcggcagcg
tggctgcgccggcggtgtgccgccccagcggctcgcggtggacgccgacgccggagcagatcaggatg
ctgaaggagctctactacggctgcggcatccggtcgcccagctcggagcagatccagcgcatcaccgc
catgctgcggcagcacggcaagatcgagggcaagaacgtcttctactggttccagaaccacaaggccc
gcgagcgccagaagcgccgcctcaccagcctcgacgtcaacgtgcccgccgccggcgcggccgacgcc
accaccagccaactcggcgtcctctcgctgtcgtcgccgccgccttcaggcgcggcgcctccctcgcc
cacccctcggcttctacgccgccggcaatggcggcggatcggctgtgctgctggacacgagttccgact
ggggcagcagcggcgctgccatggccaccgagacatgcttcctgcaggactacatgggcgtgacggac
acgggcagctcgtcgcagtggccacgcttctcgtcgtcggacacgataatggcggcggccgcggcgcg
ggcggcgacgacgcgggcgcccgagacgctccctctcttcccgacctgcggcgacgacggcggcagcg
gtagcagcagctacttgccgttctggggtgccgcgtccacaactgccggcgccacttcttccgttgcg
atccagcagcaacaccagctgcaggagcagtacagcttttacagcaacagcaacagcacccagctggc
cggcaccggcaaccaagacgtatcggcaacagcagcagcagccgccgccctggagctgagcctcagct
catggtgctccccttaccctgctgcagggagtatgtgatcagtgagacctacc

Bsa1-C-MoCRE-D-Bsa1 (1249bp)

ggtctcaggctccatgtccaacctgctcacggttcaccagaaccttccggctcttccagtggacgcga
cgtccgatgaagtcaggaagaacctcatggacatgttccgcgacaggcaagcgttcagcgagcacacc
tggaagatgctgctctccgtctgccgctcctgggctgcatggtgcaagctgaacaacaggaagtggtt
ccccgctgagcccgaggacgtgagggattaccttctgtacctgcaagcgcgaggtttgtttctgcttc
tacctttgatatatatataataattatcattaattagtagtaatataatatttcaaatattttttca
aaataaaagaatgtagtatatagcaattgcttttctgtagtttataagtgtgtatattttaatttata
acttttctaatatatgaccaaaacatggtgatgcctaggtctggcagtgaagaccatccagcaacacc
ttggacaactgaacatgcttcacaggcgctccggcctcccgcgccccagcgactcgaacgccgtgagc
ctcgtcatgcgccgcatcaggaaggaaaacgtcgatgccggcgaaagggcaaagcaggccctcgcgtt
cgagaggaccgatttcgaccaggtccgcagcctgatggagaacagcgacaggtgccaggacattagga
acctggcgttcctcggaattgcatacaacacgctcctcaggatcgcggaaattgcccgcattcgcgtg
aaggacattagccgcaccgacggcggcaggatgcttatccacattggcaggaccaagacgctcgtttc
caccgcaggcgtcgaaaggccctcagcctcggagtgaccaagctcgtcgaacgctggatctccgtgt
ccggcgtcgcggacgacccaaacaactacctcttctgccgcgtccgcaagaacggggtggctgcccct
agcgccaccagccaactcagcacgagggccttggaaggtattttcgaggccacccaccgcctgatcta
cggcgcgaaggatgacagcggtcaacgctacctcgcatggtccgggcactccgcccgcgttggagctg
ctagggacatggcccgcgccggtgtttccatccccgaaatcatgcaggcgggtggatggacgaacgtg
aacattgtcatgaactacattcgcaaccttgacagcgagacgggcgcaatggttcgcctcctggaaga
tggtgactgatcagtgagacc

Bsa1-C-framing seq-ATG-loxP- ZmNLS-mScarlet-Stop*-D-Bsa1 (no ATG) (856bp)

ggtctcaggctccatgataacttcgtatagcatacattatacgaagttatcccctaccgaggaaagag
tgaggaaaagaaaggaatccaatagagaatcagccagacgctcgagatacaggaaagccgctcacctg
aaagaactggtgagcaagggcgaggcagtgatcaaggagttcatgcggttcaaggtgcacatggaggg
ctccatgaacggccacgagttcgagatcgagggcgagggcgagggccgcccctacgagggcacccaga
ccgccaagctgaaggtgaccaagggtggccccctgcccttctcctgggacatcctgtcccctcagttc
atgtacggctccagggccttcaccaagcaccccgccgacatccccgactactataagcagtccttccc
cgagggcttcaagtgggagcgcgtgatgaacttcgaggacggcggcgccgtgaccgtgacccaggaca
cctccctggaggacggcaccctgatctacaaggtgaagctccgcggcaccaacttccctcctgacggc
cccgtaatgcagaagaagacaatgggctgggaagcgtccaccgagcggttgtaccccgaggacggcgt
gctgaagggcgacattaagatggccctgcgcctgaaggacggcggccgctacctggcggacttcaaga
ccacctacaaggccaagaagcccgtgcagatgcccggcgcctacaacgtcgaccgcaagttggacatc
acctcccacaacgaggactacaccgtggtggaacagtacgaacgctccgagggccgccactccaccgg
cggcatggacgagctgtacaagtgatcagtgagacc

Bsa1-A-pZmGLB1-C-Bsa1 (1459bp)

ggtctcaacctgcttgccgagtgccatccttggacactcgataaagtatatttatttttttatttt
gccaaccaaacttttgtggtatgttcctacactatgtagatctacatgtaccattttggcacaatta
catatttacaaaaatgttttctataaatattagatttagttcgtttatttgaatttcttcggaaaatt
cacatttaaactgcaagtcactcgaaacatggaaaaccgtgcatgcaaaataaatgatatgcatgtta
tctagcacaagttacgaccgatttcagaagcagaccagaatcttcaagcaccatgctcactaaacatg
accgtgaacttgttatctagttgtttaaaaattgtataaaacacaaataaagtcagaaattaatgaaa
cttgtccacatgtcatgatatcatatatagaggttgtgataaaaatttgataatgtttcggtaaagtt
gtgacgtactatgtgtagaaacctaagtgacctacacataaaatcatagagtttcaatgtagttcact
cgacaaagactttgtcaagtgtccgataaaaagtactcgacaaagaagccgttgtcgatgtactgttc
gtcgagatctctttgtcgagtgtcacactaggcaaagtctttacggagtgttttcaggctttgacac
tcggcaaagcgctcgattccagtagtgacagtaatttgcatcaaaaatagctgagagatttaggcccc
gtttcaatctcacgggataaagtttagcttcctgctaaactttagctatatgaattgaagtgctaaag
tttagtttcaattaccaccattagctctcctgtttagattacaaatggctaaaagtagctaaaaaata
gctgctaaagtttatctcgcgagattgaaacagggccttaaaatgagtcaactaatagaccaactaat

tattagctattagtcgttagcttctttaatctaagctaaaaccaactaatagcttatttgttgaatta
caattagctcaacggaattctctgtttttctataaaaaaagggaaactgcccctcatttacagcaaa
ttgtccgctgcctgtcgtccagatacaatgaacgtacctagtaggaactcttttacacgctcggtcgc
tcgccgcggatcggagtcccaggaacacgacaccactgtgtaacacgacaaagtctgctcagaggcgg
ccacaccctggcgtgcaccgagccggagcccggataagcacggtaaggagagtacggcgggacgtggc
gacccgtgtgtctgctgccacgcagccttcctccacgtagccgcgcggccgcgccacgtaccagggcc
cggcgctggtataaatgcgcgctacctccgctttagttctgcatacagtcaacctaacacacccgagc
atatcacagtgacagacacgggctcgagacc

Bsa1-A-pZmAXIG1-C-Bsa1 (1459bp)

ggtctcaacctaggcgacccatcgctgctttgtctacatcatgttcttcatcatcctccccaggcgac
gcgtgctgctgttcttattcagactaccgttcgagtgactgcatggcgtacatctttctgcatcgact
ttgtacggctacatcgaacatatacacgagatgtctcgtgtgaatagagtcactaatgccttaagcat
cggttactccgtagggtacattctgttcttcttatttgtgcatatttttattgttgtttactgattat
acgagtagttatacatacatgcacatacatatcatcacatatatcacaatatttttctaaattaaatt
aaaactaaaaatgactaaatttctaacaccaacgacattgtaatgttttctccaacaactttacctat
tctacattgttctatttcgaatttcactctataaacaacatagtctacaatggaaaacagtgctttgt
acgactatatacgcgatgtgtggctacaacataagacaatatagtcgtttgaagattgaacctatata
tcggtacggttaatccgtctatgtacgtgggcatgacgaacacccgtgataacgaaggattaacgtgc
acaatcataaatccaaagtaggagcggtgcatgatgagaatcgctctcagtactcgacataatgaacc
ttacgaggtacaacaggcaggcaggcagggaccaggggccgcctttatttcaggctcgctggccccac
gggcgtgctgcgtgcacgaagggcactaccccaacctctcaccgaaaccgcgctggatcggcaaatc
aaacgaggtggtgccccgtgcccactctccacgtccacggcaccatccctctgcagccgctcaccagc
catgccgtgtcgcggaacggcacaaccacccccaacccactcacgaaaccccgtcccggccgtgcccg
tgtcggtccgcgctcggcaacgaggcggcccgcgctgctgagtcccctggacacccgacaccctgtcg
gccctttgtttattcatcccgaaatctcatctgcccccacggccgactgcgctgcgccgcccggatat
atatacccatcgttatcgatcgatcgatcgcgtcactcacgggtagctcatggtcgagcgtagcatgc
aggaacttatttgccgtgcgctcccagctctccgctcgcgtgccttccagtctgtctcacactagctg
ctgtgggacgatcgaagtgggtgtgtcagctagctagctgcgccgtgaccacgcacatgaccgcagtg
cgcgcggggctgatcaagggaaagtgatccggctcgagacc

Bsa1-A-pZmPLTP-C-Bsa1 (1459bp)

ggtctcaacctccctagctccctgcggctgttacgcggtcccccatcaatcttctgttcttgcggttg
tagcctgtgtaacagtgctagagtatgtatgataaataggttttaagtctgcttacatgacatttttt
attgtggaagagacatataaaaattagagagagtggttctcatgcaacggcggacggcccggtgctaa
aagagcttcaagacaaaataatgaaacaggaagagagtagatttatctaagagccaactttattatat
gaatgtgtttattgttggctttagatgatatggtaaggagttagagctaataatagataggctctatt
attattattattaattaaactcgctctaaggaggaaagtgggaggaagggacgaggacgatgactact
ggaagcatcgtgcatggatgatggatgtggtgtctcttaatgtaggtggccggaggatgtacgtgtta
attgcgcgataagcactcagatccaaccgcaaactacctccacactgacacactgatagagagaaaga
gagagctccgacgactgccgccgcagatgagccacgtacgtatacgacgtctgccggccggctcaggc
tgccgccatcaccctgctcgaaagtcgcgttaggcggcgccagctacataggagtatctagtctagcc
agttagtatactactactgcgctgatgatgaattaactctgcatagatactgtacttgcctccctcca
acacccaaccacctcctgctcggctcttaataacttggacacggatcgatgccatccaaggaagaaca
cgacgacgacgacggaacatccaccatgcaagcttgcatccatacgccgatacgcgtgcatccatcca
tccaccattatttccattttccaccgatcacacgtacacaggcctatttaaggagcgacatcccactg
caactctcctcaccactcatcaccagctagctctagcaaagcacttgccatctaccgaccgccgcatt
ccaaacagcccgacgagctagcagagcggcaggcacctccctcctcaaggaacggctcgagacc

30bp-EcoRV-pSbALS-HRA-tStPinII-EcoRV-30bp (3185bp)

cctctagaggatccccgggtaccgagctcggatatctactttaacgcctctaaccaacacccctttat
ctttataaggaacaataaacagaatttgccccactgttctaaatcacctaataatatccccagctaaa
aacaataaaggtttcctagaattaagacaagcatgactgttcctccaggagggtttggaacattgttg
cagtcttgcagatacgggcgaagggtgagaaacagagcggagggctggaggtgacctcggtagtcgac
gccggagttgagcttgacaacgacggggcggcccctgatggacttgaggaagtccgatggcgtctaca
ccgtcccgccggcgcccgaggcgggcctgtcgctgccgccgccgccgctgctcatcttgcgcgctgtg
cccccggcggtgtccctgtgttgcggatcgcgggtgggccaggtggatgcgagggcgacccgtttgga
ctccggccggagccgccggatccctggtcggtgtcagtgccgtttactctgggccgcacgtgtcagta
ccgtctgtagatgacaacaacccgtcgtccacagtcatgtccaaaatatcctttcttctttttttttcg
attcggatatgtatcttcctttttttttttccaaaaatcttcttgacgcaccagcgcgcacgtttgtgg
taaacgccgacacgtcggtcccacgtcgatagaccccacccaccagtgagtagcgtgtacgtattcgg
gggtgacggacgtgtcgccgtcgtcttgctagtcccattcccatctgagccacacatctctgaacaaa
aaaaaggagggaggcctccacgcacatcccccctccgtgccacccgccccaaaccctcgcgccgcctcc
gagacagccgccgcaaccatggccaccgccgccgccgcgtctaccgcgctcactggcgccactaccgc
tgcgcccaaggcgaggcgccgggcgcacctcctggccacccgccgcgccctcgccgcgcccatcaggt
gctcagcggcgtcacccgccatgccgatggctcccccggccaccccgctccggccgtggggacccacc
gagccccgcaagggtgctgacatcctcgtcgagtccctcgagcgctgcggcgtccgcgacgtctttgc
ctaccccggcggcgcgtccatggagatccaccaggcactcacccgctcccccgtcatcgccaaccacc
tcttccgccacgagcaaggggaggcctttgccgcctccggctacgcgcgctcctcgggccgcgtcggc
gtctgcatcgccacctccggccccggcgccaccaacctagtctccgcgctcgccgacgcgctgctcga
ttccgtccccatggtcgccatcacgggacaggtggcgcgacgcatgattggcaccgacgccttccagg
agactcccatcgtcgaggtcacccgctccatcaccaagcacaactacctggtcctcgacgtcgacgac
atcccccgcgtcgtgcaggaggctttcttcctcgcctcctctggtcgaccagggccggtgcttgtcga
catccccaaggacatccagcagcagatggcggtgcctgtctgggacaagcccatgagtctgcctgggt
acattgcgcgccttcccaagcccccctgcgactgagttgcttgagcaagtgctgcgtcttgttggtgaa
tcgcggcgccctgttctttatgtgggcggtggctgcgcagcatctggtgaggagttgcgacgctttgt
ggagctgactggaatcccggtcacaactactcttatgggcctcggcaacttccccagcgacgacccac
tgtctctgcgcatgctaggtatgcatgggacggtgtatgcaaattatgcagtggataaggccgatctg
ttgcttgcacttggtgtgcggtttgatgatcgcgtgacagggaagattgaggcttttgcaagcagggc
taagattgtgcacgttgatattgatccggctgagattggcaagaacaagcagccacatgtgtccatct
gtgcagatgttaaacttgctttgcagggcatgaatgctcttcttgaaggaagcacatcaaagaagagc
tttgactttggctcatggaacgatgagttggatcagcagaagagggaattccccttgggtataaaac
atctaatgaggagatccagccacaatatgctattcaggttcttgatgagctgacgaaaggcgaggcca
tcatcggcacaggtgttgggcagcaccagatgtgggcggcacagtactacacttacaagcggccaagg
cagtggttgtcctcagctggtcttggggctatgggatttggtttgccggctgctgctggtgcttctgt
ggcaaacccaggtgtcactgttgttgacatcgatggagatggtagctttctcatgaacgttcaggagc
tagctatgatccgaattgagaacctcccagtgaaggtctttgtgctaaacaaccagcacctggggatg
gtggtgcagttggaggacaggttctataaggccaacagagcgcacacatacttgggaaacccagagaa
tgaaagtgagatatatccagatttcgtgacgatcgccaaagggttcaacattccagcggtccgtgtga
caaagaagaacgaagtccgcgcagcgataaagaagatgctcgagactccagggccgtacctcttggat
ataatcgtcccacaccaggagcatgtgttgcctatgatccctagtggtggggctttcaaggatatgat
cctggatggtgatggcaggactgtgtactgactagctagtcagttaacctagacttgtccatcttctg
gattggccaacttaattaatgtatgaaataaaaggatgcacacatagtgacatgctaatcactataat
gtgggcatcaaagttgtgtgttatgtgtaattactagttatctgaataaaagagaaagagatcatcca
tatttcttatcctaaatgaatgtcacgtgtctttataattctttgatgaaccagatgcatttcattaa
ccaaatccatatacatataaatattaatcatatataattaatatcaattgggttagcaaaacaaatct
agtctaggtgtgttttgcgaagatatccaattcggcgttaattcagtacattaaaaa

Table S4. Plant media components. All components are on a per liter basis of MiliQ H_2O. ([1]) Added after autoclave; ([2]) added right before use; ([3]) adjusted to recommended pH before adding gelling agent; ([4]) Murashige and Skoog (1962); ([5]) Duchefa (Haarlem, The Netherlands); ([6]) Sigma-Aldrich (Saint Louis, Missouri, USA).

Medium components	Units/L	Infection medium	Co-cultivation	Resting	Selection I	Maturation I	Maturation II	Regeneration II
**Base components**								
MS Basal Salt[4,5]	g		4.3	4.3	4.3	4.3	4.3	4.3
Chu N6-medium[5]	g	4						
**Sugar-based components**								
Sucrose	g	68.4	30	30	30	60	60	30
Glucose	g	36						
Myo-inositol[6]	mg		100	100	100	100	100	100
**Nitrogen-based components**								
L-proline[6]	mg	700	700	700	700	700	700	
L-cysteine[6]	mg		300					
Casein hydrolysate[5]	mg	1000	100	100	100			
**Buffering components**								
MES[5]	g			0.5	0.5			
**Antibiotics**								
Cefotaxime[1]	mg			100	100	100	100	
Vancomycine[1]	mg			100	100	100		
Acetosyringone[2,6] (AS)	µM	100	100					
**Hormones and other inorganic compounds**								
Dicamba[1,6]	mg	3,32	3.32	3.32	3.32			
$AgNO_3$[1,6]	mg		15.3	15.3	15.3	15.3		
$CuSO_4$	mg					1.3	1.3	
IAA[1,6]	mg					1	0.1	
Thidiazuron[1,6]	mg					0.1		
ABA[1,6]	mg					0.1	0.1	
BAP[1,6]	mg					1		
Zeatin[1,6]	mg					0.5	0.5	
**Vitamins**								
MS-vitamines[1] (1000x stock)	mL		1	1	1	1	1	1
**Selecting agents**								
Phosphinothricin[1]	mg				1.5	6	6	6
Imazapyr[1]	mg				0.1	0.1	0.1	0.05
**Gelling agents**								
Gelrite[5]	g		3			3	3	3
Agar[6]	g			8	8			
pH[3]		5.2	5.2	5.8	5.8	5.8	5.8	5.8

Table S5. Overview of tissue culture procedure.

	Co-cultivation	*Resting*	*Selection I*	*Maturation I*	*Maturation II*	*Regeneration II*
Incubation time	3 days	6-7 days	7 days	14 days	14 days	14 days
Temperature	20-21°C	25.3°C	25.3°C	25.3°C	25.3°C	25.3°C
Light conditions	Dark	Dark	Dark	Dark	80-100 $\mu E.m^{-2}.s^{-1}$ 16:8h	80-100 $\mu E.m^{-2}.s^{-1}$ 16:8h
Plate/Box	Petri dish, 100/20 mm	Petri dish, 100/20 mm	Petri dish, 100/20 mm	Petri dish, 145/20 mm	Vitro-vent container, 107/94/96 mm	Vitro-vent container, 107/94/96 mm
Plates/Boxes per liter medium	30	30	30	10	8	8
Embryos/Calli per plate/box	50	50	26	32	4-10 (depending on size)	4-10 (depending on size)

Table S6. Regeneration of B104 using different tissue culture media. Embryos from same ears were divided over the two methods in each experiment and each experiment started at same day.

	Method 1 (no selection)			Method 2 (no selection)		
Experiment	Calli to Regeneration II medium	Calli forming plantlets	%	Calli to Regeneration II medium	Calli forming plantlets	%
1	17	1	5.9	25	25	100
2	44	8	18.2	8	8	100
3	18	0	0	8	8	100
4	8	0	0	4	4	100

TC, tissue culture. For each experiment, embryos form the same ears were split between both TC media. No selection agent was used.

Table S7. Escape rate using method 2 with PPT selection

Construct	Plantlets tested	Expressing BAR	Escape rate (%)
1	41	11	73
2	5	1	80
3	21	12	43
4	27	10	63
5	33	14	58
6	32	16	50
7	37	11	70
8	44	13	70
9	52	13	75
10	78	23	71
11	53	20	62
12	44	13	70
Average			**65**

Table S8. Transformation experiments using HRA

		IE used	Indep. transgenics	%
Experiment 1	pRA-GUS	212	0	0
	pLAPAU14	228	14	6.14
Experiment 2	pRA-GUS	216	2	0.93
	pLAPAU14	202	31	15.35
	pLAPAU16	216	11	5.09

*, presence of HRA confirmed by PCR

Table S9 Copy number analysis using ddPCR. Copy number estimation of ten independent T0 plants (primary transformants) transformed with either the pLAPAU14 or the pLAPAU16 vector. Primers were designed to amplify p*ZmPLTP*::*ZmBBM* (BBM) , t*StPinII* (HRA terminator) and *aadA* (backbone marker).

pLAPAU14					pLAPAU16				
T0 ID	*BBM*	*HRA*	*aadA*	Fert.	T0 ID	*BBM*	*HRA*	*aadA*	Fert.
A	7,1	6,0	5,1	N	A	2,5	1,8	3,5	Y
D	3,1	1,9	1,1	N	B	1,4	2,1	0,3	Y
E	2,1	3,0	2,1	N	C	1,9	2,1	0,1	Y
F	4,9	4,0	4,9	N	D	0,7	1,9	0,2	Y
G	2,2	2,1	3,6	N	E	0,0	2,0	0,1	Y
H	2,1	1,8	0,2	N	F	0,7	1,0	1,0	N
I	3,2	3,1	5,0	N	G	1,8	2,0	3,1	Y
J	2,0	2,0	0,1	N	H	1,0	1,0	1,1	Y
K	3,9	3,0	2,0	N	I	2,1	1,7	0,2	Y
M	2,2	2,1	1,1	N	J	0,5	0,9	0,1	Y
Av.	**3,3**	**2,9**	**2,5**		Av.	**1,3**	**1,6**	**1,0**	

ID, identifier; Av., average; Fert., fertility (Yes/No)

Table S10. CRISPR/Cas9 *VYL* editing in T0 plants. T0 regenerants were sampled and the targeted *VYL (Chr.9_ClpP5)* or off-target *Chr.1_ClpP5* locus PCR amplified and sequenced using Sanger sequencing. Editing was analyzed using ICE and the percentage of editing in each sample is estimated, the knockout score indicates the percentage of KO alleles, the frequency of each detected allele is given for the given genotype. The R^2-value is a goodness-of-fit measure for the ICE analysis. Capital letters indicate plants derived from the same immature embryo.

pBbm42GW7-pOsU6-sgRNA(VYL)-pZmUBIL-Cas9-G7t					
**Locus**	**Plant number**	**Indel%**	**KO score %**	**R^2 model fit**	**Genotype**
Chr.9_ClpP5	452-227-Aa	93	93	0,95	0 (2%), +1 (93%)
	452-227-Ab	94	94	0,94	+1 (94%)
	452-227-Ad	89	89	0,89	-2 (45%), +1 (44%)
	452-227-B	95	72	0,95	+1 (65%), -3 (22%), -1 (7%)
	455-227-Aa	98	48	0,98	-5 (48%), -9 (50%)
	455-227-Ab	97	50	0,97	+1 (50%), -9 (47%)
	455-227-Ac	97	55	0,97	+1 (53%), -9 (42%), -14 (2%)
	455-227-Ad	97	97	0,97	+1 (45%), -11 (52%)
	455-227-B	7	7	0,99	0 (92%), -4 (4%), +1 (3%)
	455-227-Ca	99	99	0,99	+1 (99%)
	455-227-Cb	83	83	0,83	+1 (78%), -4 (5%)
Chr.1_ClpP5	452-227-Aa	0	0	1	0
	452-227-Ab	0	0	1	0
	452-227-Ad	0	0	1	0
	452-227-B	0	0	1	0
	455-227-Aa	0	0	1	0
	455-227-Ab	0	0	1	0
	455-227-Ac	0	0	1	0
	455-227-Ad	0	0	1	0
	455-227-B	0	0	1	0
	455-227-Ca	0	0	1	0
	455-227-Cb	0	0	1	0
pLAPAU17-sgRNA(VYL)					
**Locus**	**Plant number**	**Indel%**	**KO score %**	**R^2 model fit**	**Genotype**
Chr.9_ClpP5	668-357-A	67	5	0,67	-6 (61%), -17 (3%), -29 (1%), -10 (1%), -6 (1%)
	668-357-B	94	94	0,94	+1 (46%), -4 (48%)
	668-357-C	96	48	0,96	-3 (48%), -2 (48%)
	668-357-D	94	91	0,94	+1 (36%), -8 (54%), -3 (3%), +2 (1%)
	668-357-E	94	94	0,94	+1 (54%), +2 (37%), -28 (3%)
	668-357-F	95	95	0,95	+1 (46%), -4 (49%)
	668-357-G	92	92	0,92	-11 (46%), -2 (46%)
	668-357-H	94	94	0,94	+1 (45%), -4 (49%)
Chr.1_ClpP5	668-357-A	0	0	0,99	0 (99%)
	668-357-B	23	23	0,97	0 (74%), +1 (23%)
	668-357-C	50	50	0,91	0 (41%), +1 (47%), +2 (3%)
	668-357-D	43	43	0,93	0 (50%), -13 (36%), +1 (7%)
	668-357-E	16	16	0,96	0 (80%), +1 (11%), -2 (5%)
	668-357-F	29	29	0,97	0 (68%), +1 (29%)
	668-357-G	19	19	0,97	0 (78%), +1 (19%)
	668-357-H	15	15	0,78	0 (63%), -2 (7%), +1 (4%), -2 (4%)
