# Supplementary figures and images for: Optimized Transformation and Gene Editing of the B104 Public Maize Inbred by Improved Tissue Culture and Use of Morphogenic Regulators
Source: Front Plant Sci. 2022 Apr 22;13:883847. doi: 10.3389/fpls.2022.883847 (PMC9072829; doi:10.3389/fpls.2022.883847)

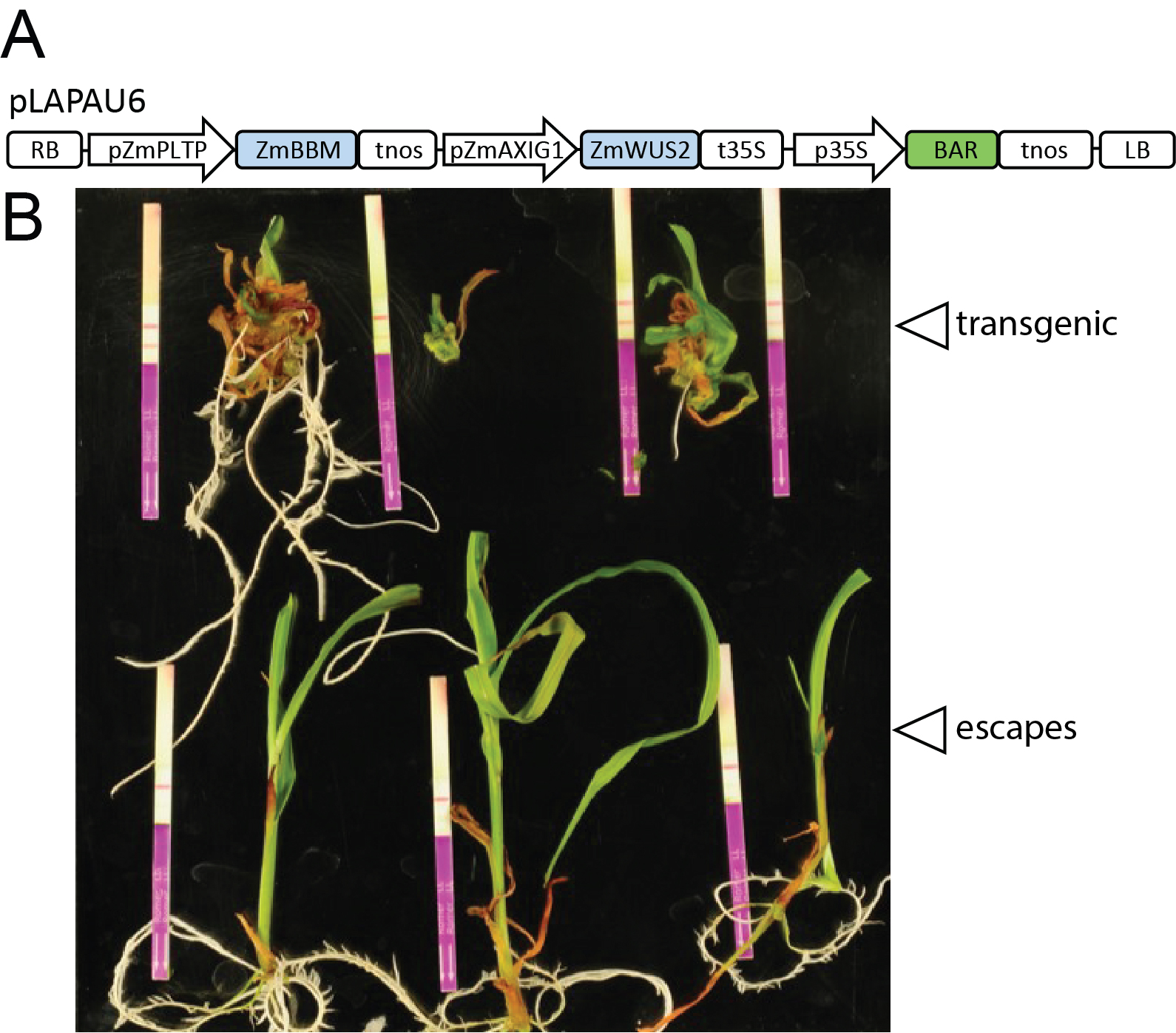

Supplement: Supplementary Figure S1 — Expression of morphogenic regulators causes developmental defects. [file Image_1.JPEG]
